# Supplementary figures and images for: iNOS is essential to maintain a protective Th1/Th2 response and the production of cytokines/chemokines against Schistosoma japonicum infection in rats
Source: PLoS Negl Trop Dis. 2022 May 18;16(5):e0010403. doi: 10.1371/journal.pntd.0010403 (PMC9116669; doi:10.1371/journal.pntd.0010403)

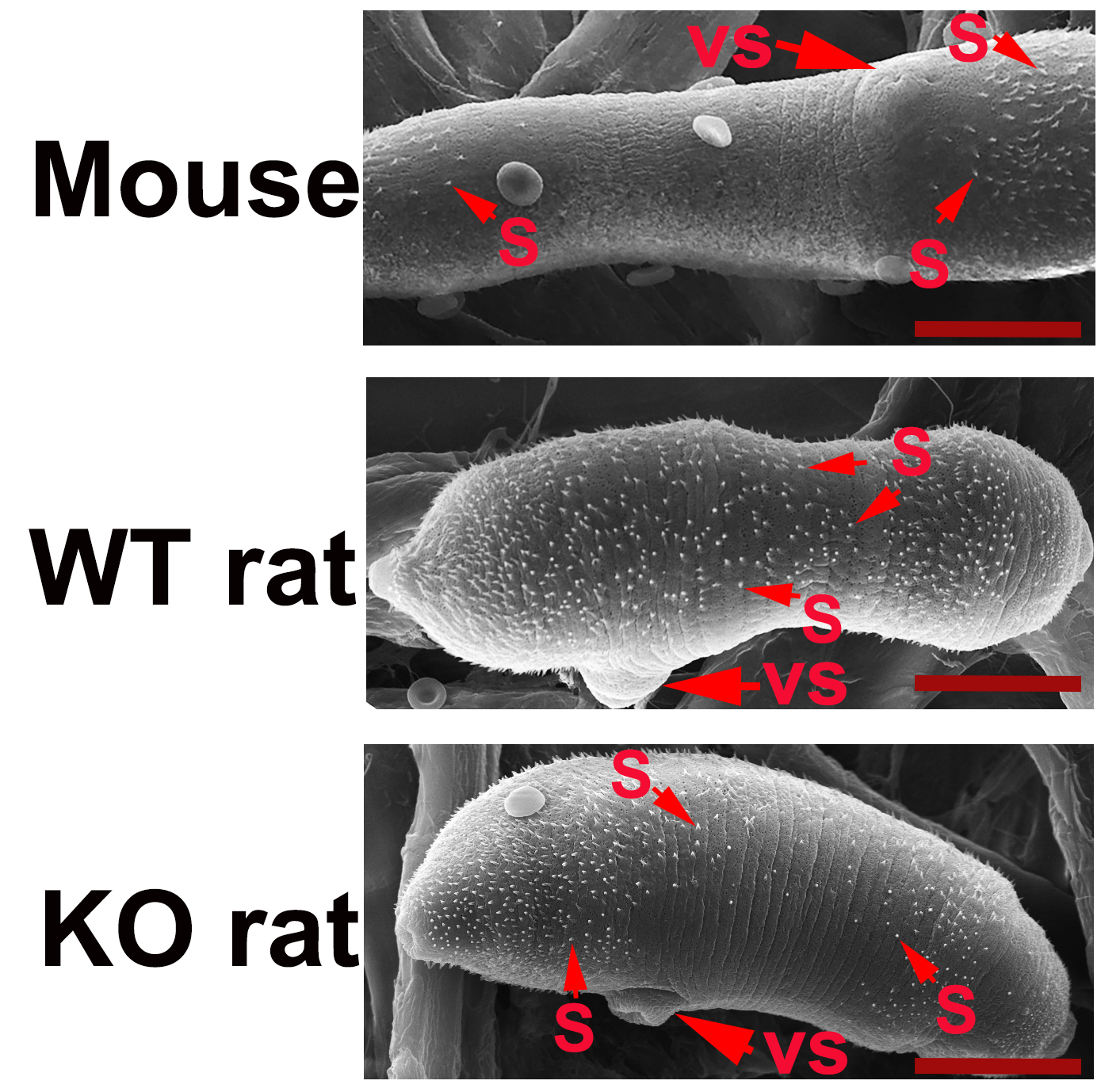

Supplement: S1 Fig — The loss of spines in the middle part of the body of schistosomula was found in mice and iNOS-KO rats, while large numbers of spines on the tegument are still present in the middle part of the body of schistosomula obtained from WT rats, suggesting parasite growth retardation. (TIF) [file pntd.0010403.s002.tif]

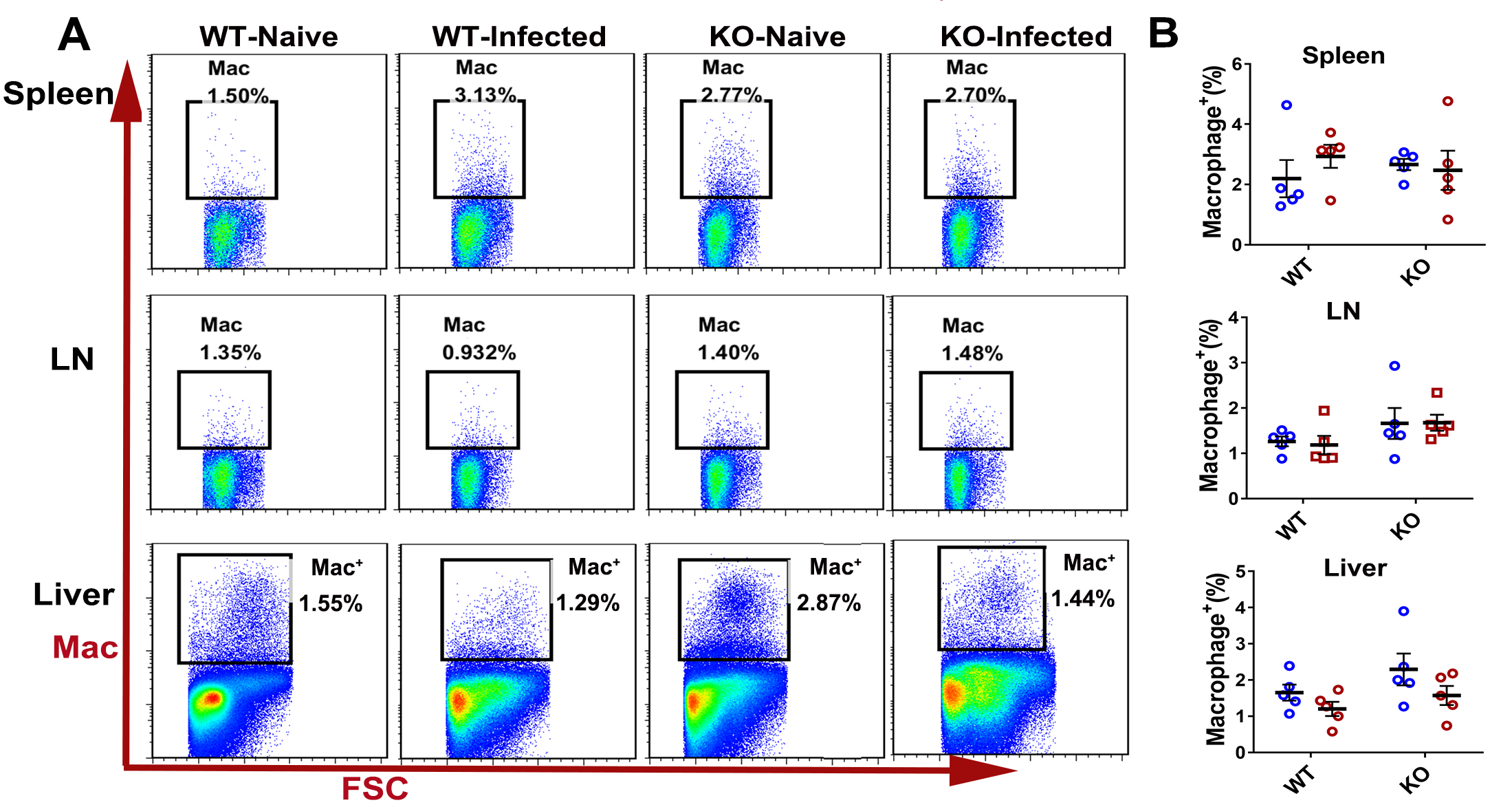

Supplement: S2 Fig — WT and KO rats were infected percutaneously with 200 S. japonicum cercariae and sacrificed at 7 weeks post-infection. (A) Representative FACS plots of macrophages in the spleens, LN, and livers. (B) Frequency of macrophages in the spleens, LN, and livers. Results for individual rats are shown and statistically significant differences are indicated. Data shown are mean ± SEM and repeated twice with similar results. n = 5 rats per group. No significant differences were found. LN = lymph node. (TIF) [file pntd.0010403.s003.tif]
