# Supplementary material for: iNOS is essential to maintain a protective Th1/Th2 response and the production of cytokines/chemokines against Schistosoma japonicum infection in rats
Source: PLoS Negl Trop Dis. 2022 May 18;16(5):e0010403. doi: 10.1371/journal.pntd.0010403 (PMC9116669; doi:10.1371/journal.pntd.0010403)

PLOS Neglected Tropical Diseases
iNOS is essential to maintain host immune activity against Schistosoma japonicum infection in rats
--Manuscript Draft--

**Manuscript Number:**	PNTD-D-21-00912
**Full Title:**	iNOS is essential to maintain host immune activity against Schistosoma japonicum infection in rats
**Short Title:**	iNOS is essential for immunity to Schistosoma japonicum in rats
**Article Type:**	Research Article
**Keywords:**	Schistosoma japonicum; rat; iNOS; resistance to infection; host immune activity
**Abstract:**	Humans and a wide range of mammals are generally susceptible to Schistosoma infection, while some rodents such as Rattus rats and Microtus spp are not. We previously demonstrated that inherent high expression levels of nitric oxide (NO), produced by inducible nitric oxide synthase (iNOS), plays an important role in blocking the growth and development of Schistosoma japonicum in wild-type rats. However, the potential regulatory effects of NO on the the immune system and immune response to S. japonicum infection in rats is still unknown. In this study, we used iNOS-knockout (KO) rats to determine the role of iNOS-derived NO in the immune system and immunopathological responses to S. japonicum infection in rats. Our data showed that iNOS deficiency led to weakened immune activity against S. japonicum infection. This was characterized by the impaired T cell responses and a significant decrease in S. japonicum- elicited Th2 responses and in cytokine and chemokine producing capability in the infected iNOS-KO rats. Unlike iNOS-KO mice, Th1-associated cytokines were also decreased in the absence of iNOS in rats. In addition, a profile of pro-inflammatory and pro-fibrogenic cytokines was detected in serum associated with iNOS deficiency. The alterations in immune responses and cytokine patterns were correlated with a slower clearance of parasites, exacerbated granuloma formation and fibrosis following S. japonicum infection in iNOS-KO rats. Furthermore, we have provided direct evidence that high levels of NO in rats can promote the development of pulmonary fibrosis induced by egg antigens of S. japonicum, but not inflammation, which was negatively correlated with the expression of TGF-β3. These studies are the first description of the immunological and pathological profiles in iNOS-KO rats infected with S. japonicum and demonstrate key differences between the responses found in mice. Our results significantly enhance our understanding of the immunoregulatory effects of NO on defensive and immunopathological responses in rats and of the broader nature of resistance to pathogens such as S. japonicum.
**Additional Information:**	
**Question**	**Response**
**Financial Disclosure**<br><br>Enter a financial disclosure statement that describes the sources of funding for the work included in this submission. Review the submission guidelines for detailed requirements. View published research articles from *PLOS NTDs* for specific examples.<br><br>This statement is required for submission and **will appear in the published article** if the submission is accepted. Please make sure it is accurate.	This work was supported by grants from the National Natural Science Foundation of China (Grant No. 81802036, J.S.; No. 81871682, Z.D.W.; http://www.nsfc.gov.cn/), the National Key R&D Program of China (No. 2020YFC1200100 and No. 2016YFC1200500, Z.D.W.; https://program.most.gov.cn/), the Natural Science Foundation of Guangdong Province, China (2020A1515010896, J.S.; http://pro.gdstc.gd.gov.cn/), the China Postdoctoral Science Foundation (No. 2018M631027 and 2019T120770, J.S.; http://jj.chinapostdoctor.org.cn/). All funds play a role in the purchase of experimental materials, the use of testing instruments, the remuneration of labor services, and so on.

Powered by Editorial Manager® and ProduXion Manager® from Aries Systems Corporation

**Unfunded studies**<br><br>Enter: *The author(s) received no specific funding for this work.*<br><br>**Funded studies**<br><br>Enter a statement with the following details:<br>• Initials of the authors who received each award<br>• Grant numbers awarded to each author<br>• The full name of each funder<br>• URL of each funder website<br>• Did the sponsors or funders play any role in the study design, data collection and analysis, decision to publish, or preparation of the manuscript?<br>• **NO** - Include this sentence at the end of your statement: *The funders had no role in study design, data collection and analysis, decision to publish, or preparation of the manuscript.*<br>• **YES** - Specify the role(s) played.<br><br>* typeset	
**Competing Interests**<br><br>Use the instructions below to enter a competing interest statement for this submission. On behalf of all authors, disclose any competing interests that could be perceived to bias this work—acknowledging all financial support and any other relevant financial or non-financial competing interests.<br><br>This statement **will appear in the published article** if the submission is accepted. Please make sure it is accurate. View published research articles from *PLOS NTDs* for specific examples.	The authors have declared that no competing interests exist.

Powered by Editorial Manager® and ProduXion Manager® from Aries Systems Corporation

**NO authors have competing interests**<br><br>Enter: *The authors have declared that no competing interests exist.*<br><br>**Authors with competing interests**<br><br>Enter competing interest details beginning with this statement:<br><br>*I have read the journal's policy and the authors of this manuscript have the following competing interests: [insert competing interests here]*<br><br>* typeset<br><br>This statement is required for submission and **will appear in the published article** if the submission is accepted. Please make sure it is accurate and that any funding sources listed in your Funding Information later in the submission form are also declared in your Financial Disclosure statement.	
**Data Availability**<br><br>Authors are required to make all data underlying the findings described fully available, without restriction, and from the time of publication. PLOS allows rare exceptions to address legal and ethical concerns. See the PLOS Data Policy and FAQ for detailed information.	Yes - all data are fully available without restriction

A Data Availability Statement describing where the data can be found is required at submission. Your answers to this question constitute the Data Availability Statement and **will be published in the article**, if accepted.<br><br>**Important:** Stating 'data available on request from the author' is not sufficient. If your data are only available upon request, select 'No' for the first question and explain your exceptional situation in the text box.<br><br>Do the authors confirm that all data underlying the findings described in their manuscript are fully available without restriction?	
**Describe where the data may be found in full sentences. If you are copying our sample text, replace any instances of XXX with the appropriate details.**<br><br>• If the data are **held or will be held in a public repository**, include URLs, accession numbers or DOIs. If this information will only be available after acceptance, indicate this by ticking the box below. For example: *All XXX files are available from the XXX database (accession number(s) XXX, XXX.).*<br>• If the data are all contained **within the manuscript and/or Supporting Information files**, enter the following: *All relevant data are within the manuscript and its Supporting Information files.*<br>• If neither of these applies but you are able to provide **details of access elsewhere**, with or without limitations, please do so. For example:	All relevant data are within the manuscript and its Supporting Information files.

Data cannot be shared publicly because of [XXX]. Data are available from the XXX Institutional Data Access / Ethics Committee (contact via XXX) for researchers who meet the criteria for access to confidential data.<br><br>*The data underlying the results presented in the study are available from (include the name of the third party and contact information or URL).*<br>• This text is appropriate if the data are owned by a third party and authors do not have permission to share the data.<br><br>* typeset	
Additional data availability information:	

Powered by Editorial Manager® and ProduXion Manager® from Aries Systems Corporation

Manuscript Click here to access/download;Manuscript;Manuscript.docx

iNOS is essential to maintain host immune activity against *Schistosoma japonicum* infection in rats

Jia Shen[1,3], Si-fei Yu[4], Mei Peng[1,3], De-Hua Lai[2], Geoff Hide[5], Zhong-Dao Wu[1,3]*, Zhao-Rong Lun[2]*

[1]Department of Parasitology and Key Laboratory of Tropical Disease Control (SYSU), Ministry of Education, Zhongshan School of Medicine, Sun Yat-Sen University, Guangzhou 510080, P.R. China.

[2] State Key Laboratory of Biocontrol, School of Life Sciences, Sun Yat-Sen University, Guangzhou 510275, P.R. China.

[3]Provincial Engineering Technology Research Center for Biological Vector Control, Guangzhou, Guangdong, 510080, China.

[4]Clinical Research Institute, The First People's Hospital of Foshan, Foshan, 528000, China.

[5]Ecosystems and Environment Research Centre and Biomedical Research Centre, School of Science, Engineering and Environment, University of Salford, Salford M5 4WT, UK.

***Correspondence:**

lsslzr@mail.sysu.edu.cn

wuzhd@mail.sysu.edu.cn

Abstract

Humans and a wide range of mammals are generally susceptible to *Schistosoma* infection, while some rodents such as *Rattus* rats and *Microtus* spp are not. We

previously demonstrated that inherent high expression levels of nitric oxide (NO), produced by inducible nitric oxide synthase (iNOS), plays an important role in blocking the growth and development of *Schistosoma japonicum* in wild-type rats. However, the potential regulatory effects of NO on the immune system and immune response to *S. japonicum* infection in rats is still unknown. In this study, we used iNOS-knockout (KO) rats to determine the role of iNOS-derived NO in the immune system and immunopathological responses to *S. japonicum* infection in rats. Our data showed that iNOS deficiency led to weakened immune activity against *S. japonicum* infection. This was characterized by the impaired T cell responses and a significant decrease in *S. japonicum*-elicited Th2 responses and in cytokine and chemokine producing capability in the infected iNOS-KO rats. Unlike iNOS-KO mice, Th1-associated cytokines were also decreased in the absence of iNOS in rats. In addition, a profile of pro-inflammatory and pro-fibrogenic cytokines was detected in serum associated with iNOS deficiency. The alterations in immune responses and cytokine patterns were correlated with a slower clearance of parasites, exacerbated granuloma formation and fibrosis following *S. japonicum* infection in iNOS-KO rats. Furthermore, we have provided direct evidence that high levels of NO in rats can promote the development of pulmonary fibrosis induced by egg antigens of *S. japonicum*, but not inflammation, which was negatively correlated with the expression of TGF-β3. These studies are the first description of the immunological and pathological profiles in iNOS-KO rats infected with *S. japonicum* and demonstrate key differences between the responses found in mice. Our results significantly enhance our understanding of the immunoregulatory effects of NO on defensive and immunopathological responses in rats and of the broader nature of resistance to pathogens such as *S. japonicum.*

Keywords：*Schistosoma japonicum;* Rat; iNOS; resistance to infection; host immune activity

Author summary

Schistosomiasis is a zoonosis that affects more than 200 million people worldwide. A wide range of mammals, including mice, are permissive hosts of *Schistosoma* and develop chronic disease characterized by egg-granuloma formation and fibrosis after infection. Rats, on the other hand, are non-permissive hosts and develop efficient immune responses to eliminate the worms. Interestingly, schistosome eggs elicit a dominant Th2 immune response within mouse hosts, whereas rats with schistosomiasis develop a significant Th2 response in the absence of available egg production. The Th2 response in rats seems to play an essential role in the protection of the host against *Schistosoma*. So far, the factors that lead to the different immune responses to *Schistosoma* infection in both hosts have not been demonstrated. In this study, our results show that an iNOS-dependent mechanism maintains the function of the immune system in rats by modulating $CD4^+$ T cell-mediated Th1/Th2-associated cytokine responses and chemokine production. Additionally, the absence of iNOS led to slow clearance of parasites, increases in the development of worms and an exacerbation of granuloma formation and fibrosis in rats. Furthermore, high levels of NO in rats can promote the development of fibrosis induced by inflammation (rapid inflammatory repair). Therefore, this study demonstrates that the difference in iNOS levels between mice and rats is responsible for the different immune responses and outcomes induced by schistosome infection in both hosts.

Introduction

Schistosomiasis, is a zoonosis caused by the parasitic helminth *Schistosoma*. It is

an important public health problem that affects around 290 million people in 78 countries [1]. The bulk of the morbidity and mortality of schistosomiasis ultimately results from the development of a Th2-driven inflammatory response and fibrosis in the host causing by *Schistosoma* eggs trapping in tissues, such as intestines and liver (intestinal schistosomiasis) and the urinary bladder wall (urogenital schistosomiasis) [2]. A wide range of mammals, including humans, are generally susceptible/permissive hosts of *Schistosoma* and develop chronic disease characterized by egg-granuloma formation and fibrosis after infection [3, 4]. In contrast, the rat is a nonpermissive host in which the parasite does not cause typical egg granulomas in the liver of the infected host. This is because the rat is genetically resistant to *Schistosoma* infection and develops efficient immune responses to eliminate the worms [4] [5]. Typically, mice are widely used as a model of permissive host, for *Schistosoma* spp, to understand the immune responses during human schistosomiasis. During the initial stages of infection, mice display a Th1 immune response by producing high levels of Th1 cytokines (e.g., IFN-γ, interleukin (IL) -12, TNF-α), which may participate in protection against *Schistosoma* infection [6]. However, once *Schistosoma* eggs are produced at 5-6 weeks of infection, the host immune status dramatically shifts to a Th2 response, as shown by the increased production of Th2-associated cytokines IL-4, IL-5, IL-10, and IL-13 [7, 8]. The Th2 response is involved in the development of egg-granuloma formation and fibrosis and is essential for host survival by protecting against inflammatory cytokines the mediate death during acute schistosomiasis. The excretory-secretory antigens (ES) from the eggs are powerful factors that induce Th2 polarization in schistosomiasis. A particularly striking difference, in the rat model of schistosomiasis compared to mice, is the fact that a Th2 response is driven in the absence of available egg production by worm pairs [4, 9]. Therefore, it suggests that the, significantly greater, Th2 type response in rats seems to play an essential role in the protection of the host against *Schistosoma* [4]. Consequently, the different immune responses induced by

schistosomes in mice and rats may be responsible for the different outcomes of host adaptability. However, the factors that lead to the different immune responses to *Schistosoma* infection in both hosts have not been elucidated. In previous work, we demonstrated a key role of nitric oxide in resistance to schistosome infection in rats suggesting its involvement in this process [10].

Nitric oxide (NO), produced from L-arginine by three isoforms of nitric oxide synthase (NOS), exhibits a variety of physiologic functions in mammals [11]. It is reported that NO plays an important role in regulating vascular function, neurotransmission, inflammatory responses, immune function, and host defense [12, 13]. Inducible NOS (iNOS), expressed in response to proinflammatory cytokines (such as IFN-γ, TNF-a, and IL-1β) and/or microbial products (such as LPS), can rapidly produce large amounts of NO in contrast to the other two isoforms, the endothelial NOS (eNOS) and the neuronal NOS (nNOS) [12]. In previous studies, we have confirmed that the expression of iNOS and the levels of NO induced by stimulation of macrophages of rats are significantly higher than those in mice [10, 14, 15]. Furthermore, using iNOS knockout (iNOS-KO) rats, we have demonstrated that NO, inherently at high levels in rats, plays a crucial role in blocking *S. japonicum* growth, reproductive organ development, egg production, and the ability to lay fertilized eggs [10]. Furthermore, the inhibitory effect of NO acts by affecting mitochondrial respiration and energy production in this parasite [10]. Indeed, in addition to its direct cytotoxic effect on infectious pathogens in non-specific immune defense mechanisms, NO has also been found to be a potent immunoregulatory factor [16, 17]. Previously, studies have shown that NO displays a significant immunosuppressive effect by causing inhibition of T cell proliferation, regulation of T-cell function [18-20] and inducing T helper cell deviation by suppression of Th1 (and Th2) cell responses [21, 22]. Moreover, NO has also been demonstrated to influence the expression and function of many inflammatory factors [23]. Therefore, it raises the question as to whether the high expression of NO in rats affects the

development of *S. japonicum* by regulating the immune response.

The majority of previous studies on the effects of NO on the immune system were based on mouse models. However, the effects of NO on the immune system vary with concentration, host species and response to different pathogens. For example, it was reported that high concentrations of NO could suppress the expansion of Th1 cells by inhibiting IL-12 synthesis, whereas low doses of NO selectively promoted Th1 cell differentiation and had no effect on Th2 cells [21]. Furthermore, NO has been reported to preferentially down-regulate Th1-mediated immune responses in the murine system [24, 25], however, this selective inhibitory effect on Th1 responses was not found in activated human T cells [22]. Additionally, NO induces different immune responses to different pathogens in the same host. It is shown that a Th1 immune response was enhanced in iNOS-KO mice after infection with *Leishmania major* [26], whereas a gastric *Helicobacter* infection displayed a reduction of the Th1 response in iNOS-KO mice, relative to that of the WT mice [27]. Thus far, little is known in rats about the regulatory role of NO on the immune system and the outcomes of iNOS gene deficiency on the evolving immune response. It is possible that the differences in NO levels between mice and rats [14] may be linked to differences in *Schistosoma* infection-induced immune responses. Therefore, there is a need to investigate the role of NO in the regulation of the immune response that inhibits *Schistosoma* infection in rats.

In the present study, using *S. japonicum* infected iNOS-KO and wild-type rat models, we investigated the effects of iNOS-derived NO on immunity and immunopathological responses to *S. japonicum* infection in rats. Our results show that an iNOS-dependent mechanism maintains the function of the immune system in rats by modulating $CD4^+$ T cell-mediated Th1/Th2-associated cytokine responses and chemokine production. This plays a crucial role in host protective immunity against *S. japonicum* infection in rats. Thus, this study significantly enhances our understanding of the immunoregulatory effects of NO on defensive and immunopathological

responses in rats.

Results

iNOS-dependent functions are essential for host protection against *S. japonicum* infection in rats

To examine the role of iNOS against *S. japonicum* infection in rats, WT and iNOS-KO rats were infected with *S. japonicum*. As shown in Fig. 1A, the worm burden collected from the hepatic portal vein infusion of infected KO rats, compared with infected WT rats, was greater by about 1.5-fold at week 1 ($P < 0.05$) and 3-fold at week 7 post-infection ($P < 0.01$). Interestingly, although the worm load showed a slight increase in the infected KO rats at week 4 post-infection and was higher than those found at week 1 post-infection, there was no significant difference ($P > 0.05$) between the two groups. The results suggest that iNOS in rats promotes the elimination of *S. japonicum*, particularly after 4 weeks post-infection. Additionally, in contrast to the stunted parasites found in WT rats, the growth and development of *S. japonicum* in iNOS-KO rats are markedly more healthy (Fig. 1B).

Hepatic granulomatous inflammation and fibrosis is the primary cause of chronic morbidity in schistosomiasis. Strikingly, the infected KO rats developed significant granulomatous inflammation and fibrosis in the liver, particularly at the chronic time point of 12 weeks post-infection, compared with infected WT rats (Fig. 1C). Dead worms in the liver sections were clearly observed in the infected KO rats (Fig. 1C). Moreover, infected KO rats experienced significantly greater weight loss than infected WT rats, especially after 4 weeks post-infection ($P < 0.01$) (Fig. 1D). When infected with a low dose of parasites, no death was found both in WT or KO rats. However, at high doses, the survival of iNOS-KO rats was dramatically decreased and ultimately 100% of animals succumbed between days 11-34 post-infection, while no

mortality was observed in the WT control group during this period (Fig. 1E). Together, these results showed that iNOS-mediated functions are essential in host protection against *S. japonicum* infection in rats, as characterized by accelerating clearance of parasites, alleviating hepatic pathological responses and promoting host survival.

iNOS deficiency led to a decrease in the frequency of T cells and an increase in B cells in rats infected with *S. japonicum*

In order to determine whether iNOS is involved in anti-*Schistosoma* infective immunity by affecting the profile of immune cells in rats, we examined the differences in frequencies of T cells and B cells in spleens, mesenteric lymph nodes (LN), and liver cells by comparing infected iNOS-KO rats and WT rats. In comparison with WT rats, the results showed that there was a lower frequency of $CD3^+$ T cells ($P < 0.05$) and a significantly higher frequency of $CD19^+$ B cells ($P < 0.05$) both in LN and liver cells of KO rats (naïve and infected) but not in spleens (Fig. 2A and 2B). This suggested an attenuation of T cell function and increased B cell function in iNOS-KO rats. However, no significant differences were observed in the frequency of macrophages in the spleen, LN, and liver between WT rats and KO rats ($P > 0.05$) (Fig. S1). Thus, these results demonstrate the role of iNOS in maintaining the host's T-cell immune response and the immunosuppressive effect on B cells in rats during *S. japonicum*. Furthermore, it indicates that iNOS-mediated T-cell immune responses may play an important role in host protection against *S. japonicum* infection in rats.

To evaluate the role of iNOS in regulating the host's T cell immune function in rats during *Schistosoma* infection, the proportion of $CD4^+$ and $CD8^+$ T cell subsets in spleens, mesenteric LN, and livers from WT and iNOS-KO rats were analyzed. As shown in Fig. 2C and 2D, the naïve KO rat displayed a lower frequency of $CD4^+$ T

cells in the LN ($P < 0.05$) in comparison with naïve WT rats. However, there was no significant difference in the percentage of $CD8^+$ T cells in all examined tissues between the two naïve groups. After infection with *S. japonicum*, WT rats showed a decrease in the percentage of $CD4^+$ T cells in the spleen ($P < 0.05$), LN ($P < 0.05$), and liver ($P < 0.01$), and a slight increase in the percentage of $CD8^+$ T cells in the liver ($P < 0.05$). Surprisingly, an increase in the percentage of $CD4^+$ T cells and a reduction in the percentage of $CD8^+$ T cells in the spleen were found in the KO rats following infection ($P < 0.05$). Interestingly, the results found for the liver were opposite to the other tissues.

The $CD4^+/CD8^+$ ratio, an indicator for assessing the host cellular immune status, was also compared and analyzed between in WT and KO rats. The results showed that the $CD4^+/CD8^+$ ratio in the blood (1.63, $P < 0.05$), LN (2.99, $P < 0.05$), and liver (0.56, $P < 0.05$) of WT rats were significantly higher than those in KO rats (blood, 0.87; LN, 2.38; liver, 0.39), and significantly reduced in the spleen (1.19, $P < 0.05$) and liver (0.18, $P < 0.05$) following infected with *S. japonicum* (Fig.2E). Surprisingly, however, the decreased $CD4^+/CD8^+$ ratio was also not found in the blood, spleen and LN of KO rats following infection with *S. japonicum* (Fig.2E). Thus, the results reveal that NO is necessary for maintaining the host's cellular immune defense against infection in wild-type rats.

A profile of pro-inflammatory and pro-fibrogenic cytokines developed in iNOS deficient rats with schistosomiasis

To investigate the systemic immune response developed in KO rats, cytokine-producing profiles were measured in serum at different time-points post-infection with *S. japonicum*. These included the pro-inflammatory cytokines (TNF-α, IL-6, and IL-1β), Th1 cytokines (IFN-γ, IL-2), Th2 cytokines (IL-4, IL-5, and IL-13) and regulatory cytokines (IL-10). As demonstrated in Figure 3, when the iNOS gene was

ablated in rats, it developed a pro-inflammatory immune environment and Th1 immune deviation, with an increase in pro-inflammatory cytokines (TNF-α, IL-1β, and IL-6, $P < 0.05$) (Fig. 3A) and in Th1 cytokines (IFN-γ, $P < 0.05$) (Fig. 3B) in naïve KO rats in comparison with naïve WT rats (0 w post-infection). After infection with *S. japonicum*, KO rats also developed a strong pro-inflammatory response, with high production of TNF-α, IL-6, and IL-1β at all time points post-infection which reached a peak at 7 weeks post-infection. Moreover, infected KO rats displayed a marked decrease in the levels of both Th1- (IFN-γ) and Th2-associated cytokines (IL-4, IL-5) at all time points post-infection, except with IL-2 and IL-13 (Fig. 3C), both of which play an important role in granuloma and fibrosis development [26,27]. In addition, the immunosuppressive cytokine IL-10 was also significantly down-regulated in KO rats, which displayed a notable significant difference at 7 weeks post-infection between both groups (Fig. 3D). Expression of IL-17 was not detectable (data not shown). Thus, it suggests that a pro-inflammatory and pro-fibrogenic immunological environment developed with iNOS deficiency in infected rats.

iNOS expression ensures maximal development of Th2 and Th1 responses in the rat during *S. japonicum* infection

Previous studies have reported that a Th2 response was strongly activated in the absence of available egg production in the rat model of schistosomiasis and speculated that the increased Th2 response was involved in the resistance to *Schistosoma* infection [4, 9]. We also observed increased levels of the Th2-related cytokines IL-4 and IL-5 in serum from infected WT rats. Therefore, to determine whether the high levels of expression of iNOS were responsible for the elevated Th2 response in rats following infection with *S. japonicum*, Th1 and Th2 responses were compared between WT and iNOS-KO rats. Here, Th1 and Th2 responses were analyzed by the expression of IFN-γ and IL-4 in $CD4^+$ T cells from peripheral blood,

LN, and spleen from 7-week *S. japonicum*-infected animals detected by flow cytometry. As shown in Fig.4A and 4B, the frequency of $CD4^+$ T cells producing IL-4 was slightly increased in the blood ($P < 0.05$) of naïve KO rats, compared with naïve WT rats. As expected, there was a marked increase in the frequency of IL-4-producing $CD4^+$T cells in the blood ($P < 0.001$), LN ($P < 0.01$), and spleens ($P < 0.001$) of WT rats following infection with *S. japonicum*. However, although the frequency of IL-4-producing $CD4^+$ T cells slightly increased in infected KO rats, when compared to naïve KO rats, no significant differences were found in LN and spleens ($P > 0.05$) although these were lower than those found in the infected WT rats. The results suggest an impaired Th2 response in infected iNOS-KO rats. The reduction in the Th2 response did not result from an increased Th1 response because the frequency of $CD4^+$ T cells producing IFN-γ was also decreased in the blood, LN, and spleens of infected KO rats, compared with infected WT rats ($P > 0.05$) (Fig.4C and 4D). The results suggest that high expression levels of iNOS are required to sustain $CD4^+$ T cell responses in rats with schistosomiasis.

Cytokine and chemokine production in local tissues (liver) is dependent on the expression of iNOS in rats during *S. japonicum* infection

The liver is the main location of pathological changes and schistosome parasitism in rats with schistosomiasis. To evaluate the role of iNOS in regulating the chemotaxis and effector effects of immune cells involved in immunopathology and activity against *Schistosoma*, we compared the immunological characteristics of the livers of WT and KO rats. As shown in Fig.5A, local (liver) IFN-γ, IL-4, IL-10, TGF-β1 levels in infected KO rats were significantly lower than those in infected WT rats ($P < 0.05$), which was consistent with the results found for serum (Fig. 3). Surprisingly, the expression of IL-6 mRNA also showed marked reduction in infected KO rats ($P < 0.01$), in contrast to the serum which showed significantly increased

expression (Fig. 3A). Again, similar findings were confirmed by immunohistochemistry, which showed that the expression of IFN-γ, IL-10, TGF-β3, and IL-6 at the periphery of granulomas in KO rats were significantly lower than those in WT rats (Fig.5B). Furthermore, the expression of the monocyte chemoattractant protein (MCP)-1, Eotaxin, Eotaxin-2, neutrophil-activating protein (NAP)-3 (also named CXCL1), macrophage inflammatory protein (MIP)-2a (also named CXCL2) were significantly decreased in the liver of infected KO rats, indicating a decline in the ability to recruit immune cells to the inflammatory site (Fig.5A). The results demonstrate that both the cytokines and chemokines responsible for the capabilities of granuloma-associated lymphocytes were diminished in the absence of iNOS in rats. This provides evidence that the expression of iNOS is required to induce the transcription of cytokines and chemokines in the liver of rats during *S. japonicum* infection, which is, in turn, very important in the immune response against *Schistosoma*.

iNOS also participates in promoting pulmonary granuloma associated fibrotic processes, but not inflammation, in wild type rats

To determine the possible immunoregulatory effects of iNOS in formation and development of fibrosis in rats, we exploited the schistosome egg-induced pulmonary granuloma model [28]. Pulmonary granuloma associated fibrosis was compared in the WT and iNOS-KO rats on day 7 and 14 post-challenge, following intravenous injection with 15,000 live eggs. We have described previously that there were no significant differences in granuloma size in both genotypes of animals on day 7 and 14 post-challenge, although the KO rats displayed a, non-significant, slight decrease [10]. When pulmonary granuloma-associated fibrosis in WT and KO rats were compared by Masson's trichrome staining (Fig. 6A and 6B), surprisingly, compared with WT rats, the KO rats showed an average of 78% increase in blue fibrosis staining

on day 7 ($P < 0.01$), the peak of the granulomatous response [29], while displaying a slight, non-significant, reduction (27%) in collagen deposition on day 14 post-challenge ($P > 0.05$). The results showed that iNOS in rats inhibited the formation of fibrosis in the acute phase and promoted fibrosis in the chronic phase. Furthermore, in comparing the fibrotic process in KO and WT rats, WT rats displayed a slight increase in collagen deposition at day 14 compared with day 7 post-challenge. In contrast, collagen deposition was markedly decreased in KO animals at day 14, with nearly 56% less than that found at day 7 post-challenge ($P < 0.01$) (Fig. 6B). In addition, fibrosis was also examined using immunohistochemistry with alpha-smooth muscle actin antibodies (α-SMA), a marker of activated myofibroblasts, as shown in Figures 6C and 6D. Consistent with the collagen deposition by Masson's trichrome, KO rats also displayed much stronger staining for α-SMA at day 7 post-challenge and weakened at day 14 post-challenge (Figure 6C and 6D). The results showed that the fibrosis process is inhibited in KO rats at day 14 post-challenge, compared with WT rats which showed persistently elevated α-SMA expression. Therefore, these results suggest that iNOS accelerates the pulmonary granuloma-associated fibrotic process in rats. On the other hand, importantly, our results illustrated that the exacerbated granuloma-associated inflammation and fibrosis in the liver of iNOS-KO rats were attributed to the increased worm burden, egg load, and viability, but not the effects of iNOS on pathology.

To dissect the possible mechanisms for iNOS-mediated promoting pulmonary granuloma-associated fibrotic process in rats, we detected the expression of cytokines at the periphery of granulomas in WT and KO models by immunohistochemistry. As shown in Fig.6E and 6F, the KO rats displayed a slight, non-significant, reduction in the expression of IFN-γ and IL-10 and a slight, non-significant, increase in the expression of IL-6 and TNF-α at day 7 and 14 in comparison with WT rats ($P > 0.05$). This is consistent with the results found in the serum of KO rats infected with *S.*

japonicum (Fig.3). Interestingly, compared with WT rats, however, the expression of TGF-β3 around pulmonary granulomas in KO rats showed nearly a 64% reduction on day 7 ($P < 0.01$) and 67% increase on day 14 post-challenge ($P < 0.05$) (Fig.6E and 6F), which is opposite to the trend observed in pulmonary granuloma associated fibrosis. To further investigate whether the expression of TGF-β3 at the periphery of granulomas in lungs was associated with the development of fibrosis in rats, we conducted a correlation analysis between the expression level of TGF-β3 around pulmonary granuloma and pulmonary granuloma associated fibrosis in WT and KO rats. Figure 6G shows that the TGF-β3 levels were negatively correlated with fibrosis quantified using Masson's Trichrome in WT and KO rats ($r = -0.55$; $P= 0.035$). No significant correlation was found between TGF-β3 levels and the expression of α-SMA at the periphery of granulomas ($r = -0.46$; $P= 0.082$) (Figure 6H). The results indicate that TGF-β3 may be involved in the iNOS-mediated pathway promoting the pulmonary fibrotic process in rats.

Discussion

The rat, a non-permissive host, develops immune responses directed at juvenile or adult *Schistosoma* that causes rapid elimination of all parasites and no typical egg-granuloma formation [4, 5], while mice (permissive hosts) do not. Interestingly, schistosome eggs elicit a dominant Th2 immune response within mouse hosts [8, 30], whereas rats with schistosomiasis develop significant a Th2 response in the absence of available egg production [4, 9]. Our previous studies have demonstrated that the expression of iNOS and the levels of NO in peritoneal macrophages of rats are significantly higher than those in mice [10, 14, 15]. In the present study, our results showed that, following infection with *S. japonicum*, iNOS gene deficiency in rats led to slow clearance of parasites, increases in the development of worms and an

exacerbation of granuloma formation and fibrosis. Importantly, *S. japonicum*-elicited a Th2 response that was significantly impaired in iNOS-KO rats. Therefore, this study demonstrates that the difference in iNOS levels between mice and rats is responsible for the different immune responses and outcomes induced by schistosome infection in both hosts.

The results presented here show that the worm burden collected from the hepatic portal vein infusion of infected iNOS-KO rats was significantly increased compared with the infected WT rats at week 1 and 7 post-infection, while no significant differences were found at week 4 post-infection. Coulson, *et al.* [31] have concluded from their results that the effector response of NO, against an embolized parasite in the lungs, acts by blocking further migration rather than by direct cytotoxic killing. Therefore, in our study, the elevated worm load, at week 4, in WT rats may be attributed to the parasites that remained in the lungs having reached the liver. This is due to the migration of *S. japonicum* from the lung to the liver being inhibited by NO in WT rats. This inference was further supported by the observations that the spines on the tegument in the middle part of the body of the schistosomula from iNOS-KO rats were much reduced compared to those found in WT rats (Fig. S2), that the loss of spines contributes to reducing the resistance during their migration [32]. Furthermore, NO in rats promotes the clearance of *S. japonicum* mainly after 4 weeks post-infection, such that the worm load decreased most obviously at 7 weeks after infection, which implies that it is in the liver rather than in the lungs that NO is effective in eliminating the parasite. This opinion is supported by the results reported by Wilson *et al.* [31] that NO was not the major agent causing the pulmonary effector response to eliminate *S. mansoni* in mice exposed to the radiation-attenuated vaccine. It is worth noting that the iNOS-KO rats died at a significantly accelerated rate, with all animals succumbing within 5 weeks infected with a high dose of parasites, compared with WT rats. The significantly increased mortality may attribute to the increased parasite burden and development-mediated inflammation, as demonstrated

by the significant increase in pro-inflammatory cytokines (TNF-α, IL-6, and IL-1β) levels in the serum of infected iNOS-KO rats within 5 weeks post-infection.

Based on the results, we deduce that *S. japonicum*–infected iNOS-KO rats are immunocompromised and unable to clear the infection. Thus, we first examined whether the generation of immune cells was affected by iNOS deficiency. Our results showed that iNOS deficiency in rats led to a reduction in the frequencies of T cells and an increase in B cell numbers in mesenteric lymph node and livers, but having no effects on macrophages. These results demonstrate that the expression of iNOS plays an important role in maintaining the host T-cell immune response and inhibiting B cell populations in rats with schistosomiasis. T cells play an essential role in the immune response to schistosomiasis. It has been reported that the preferential proliferation of T cells in the draining lymph nodes of mice exposed to *S. mansoni* is central to the induction of protective immunity [33, 34]. In contrast, the role of B cells in the process of schistosome infection seems to be less important. Ferru *et al.* [35] demonstrated that B lymphocytes were not essential for the development of the general immune response towards *S. mansoni* in the mouse, they showed that the absence of B cells in mice did not affect the adult worm and egg burdens in comparison to control groups. Thus, it indicates that iNOS-mediated T-cell immune responses may play an important role in host protective immunity against *S. japonicum* in rats. $CD4^+$ and $CD8^+$ T cells are important for regulating the host's immune function against infection [36]. $CD4^+$ T cells participate in modulating the activity of the immune response mainly by regulating the secretion of cytokines and antibodies [37], and $CD8^+$ T cells take part in the cytotoxic effect [38]. The $CD4^+/CD8^+$ ratio is one of the indicators for assessing host cellular immune status, a decline in the $CD4^+/CD8^+$ ratio implies weakening immune function of the host [36]. In our study, a decrease in $CD4^+$ T cells and $CD4^+/CD8^+$ ratio was observed in naïve iNOS-KO rats compared to WT rats, which indicated a likely decline in immune response in iNOS-KO rats. Furthermore, the increase in $CD4^+$ T cells and $CD4^+/CD8^+$

ratio in iNOS-KO rats following infection with *S. japonicum* means that this parasite infection can activate and enhance the host cellular immune functions in iNOS-KO rats. A previous study has shown that immunosuppression in murine schistosomiasis may be caused by the activation of $CD8^+$ T-cell function, which suppresses the maturation of $CD4^+$ T cell subsets [39]. Thus, the results reveal that NO is necessary for maintaining the host's cellular immune defense against *S. japonicum* in wild-type rats.

Previous studies have reported that the Th2 response plays an important role in resistance to *Schistosoma* infection in rats, that the worm burden was significantly increased in rats following treatment with anti-IL-4R or anti-IL-13R antibodies and which could be associated with their ability to control the production of IgE and eosinophils [4]. In addition, previous results revealed that the immune effector mechanism against schistosomula in the lungs requires $CD4^+$ T cells capable of producing IFN-γ in mice [31, 34]. Therefore, we compared the *S. japonicum*-elicited Th1 and Th2 responses in iNOS-KO rats and WT rats. Interestingly, in infected iNOS-KO rats, the frequency of IL-4-producing $CD4^+$T cells was impaired in the mesenteric lymph nodes and spleen, and the levels of Th2-associated cytokines (IL-4 and IL-5) in the serum and liver were also markedly reduced, compared with infected WT rats. Surprisingly, the reduction in Th2 response did not result from an increased Th1 response, because the frequency of $CD4^+$ T cells producing IFN-γ was also obviously decreased in the lymph nodes, spleens, and liver of infected iNOS-KO rats comparison to the WT rats. Therefore, the results indicate that iNOS expression ensures maximal development of Th2 and Th1 responses in the rat during *Schistosoma* infection. The downregulated Th2 response and IFN-γ levels in iNOS-KO rats are not conducive to the elimination of parasites. Interestingly, these results found in infected iNOS-KO rats differ from the observations obtained from infected iNOS-KO mice, which develop significantly enhanced Th1-associated cytokine responses (IFN-γ) and lower expression of Th2-associated cytokines (IL-4, IL-5)

levels in the lungs after vaccination with attenuated *S. mansoni* [40] and, furthermore, displayed partially reduced resistance [31, 40]. Similar Th-associated cytokine response profiles were also found in the egg/IL-12-sensitized iNOS-KO mice, while there was little or no significant effect on the Th2 or Th1-type cytokine polarization during the natural course of infection with *S. mansoni* in the iNOS-KO mice *versus* WT mice, given the low expression of iNOS detected in infected mice [41]. These data indicate that iNOS activation is not essential for Th1 response development in mice. Thus, the results obtained with iNOS-deficient animals (rats and mice) accurately reflect that the difference in NO concentration in the host could lead to different levels of resistance to *S. japonicum* by regulating the immune response of the host.

Moreover, it is of note that the transcriptional levels of all cytokines and chemokines measured in the liver (the main location of schistosome parasitism in rats) of iNOS-KO rats were markedly lower than those of WT rats. Chemokines play an important role in regulating cell trafficking. When the parasites persist in a particular organ, the chemotactic signals facilitate the recruitment of macrophages, eosinophils, basophils, and T cells to form a granuloma to keep the parasites or eggs sequestered under control [42]. Therefore, the down-regulated chemokines in iNOS-KO rats are not conducive to the recruitment of inflammatory cells to the periphery of worms and therefore were unable to secrete abundant cytokines necessary for rapid elimination of *Schistosoma*. These results demonstrate that both the cytokine and chemokine producing capabilities are ensured by the expression of iNOS in rats. This notion is consistent with the previous findings in iNOS-KO mice that the size of inflammatory foci around schistosomula in the lungs was diminished [40]. In addition, a proinflammatory immune environment was developed in naïve iNOS-deficient rats, with an increase in TNF-α, IL-1β, and IL-6 expression, compared with naïve WT rats. Previous studies have demonstrated that pro-inflammatory stimuli could promote schistosome development and exacerbate infection (44). A similar observation was

also demonstrated by Riner *et al* (45), they revealed that repeated administration of an endogenous inflammatory stimulus could restore schistosome development in recombination activating gene-deficient ($RAG^{-/-}$) mice, which fail to induce liver inflammation and necrosis after infection with *S. mansoni*. In addition, some studies showed that TNF could indirectly stimulate schistosome development by binding to the TNF receptor and TLR signaling pathway (46-49). Thus, based on our findings, we demonstrate that the high levels of expression of NO in rats can inhibit the development of *S. japonicum* by regulating the host immune system.

The role of iNOS in fibrosis is highly controversial, with some studies showing pathogenic [43-45] and protective [41, 46] roles for this enzyme. Notably, in our present study, the increased fibrosis in the liver of infected iNOS-KO rats was attributed to the marked increased egg production and the viability of parasite eggs [10], which formed larger sized granulomas and developed into more severe fibrosis in comparison to infected WT rats. Therefore, we could not determine the protective effect of NO on fibrosis in rats. With the schistosome egg-induced pulmonary granuloma model, interestingly, we found that iNOS played different roles during acute versus chronic stages. Pulmonary granuloma-associated fibrosis was inhibited at the acute stage (7 days post-challenge) by the expression of iNOS in rats, while such protection was completely abolished during the chronic stage (14 days post-challenge). In other words, these results suggest that the expression of iNOS accelerates the pulmonary granuloma-associated fibrotic process in rats. These observations were consistent with the results reported in allergen exposure-induced pulmonary fibrosis models that fibrosis was completely absent in iNOS-KO mice when using the chronic protocol [47]. The reason for this result may be that the iNOS gene deletion caused a failure to induce the infiltration of fibroblasts and collagen production in the chronic stage. While the presence of iNOS promoted the development of fibrosis, induced by inflammation, and suggests a possible cause of rapid inflammatory repair in the lungs and liver of rats with schistosomiasis. Studies

in a wide range of experimental models have demonstrated the profibrotic actions of TGF-β1 [48-51]. In contrast, some experimental studies indicated that TGF-β2 and TGF-β3 can exert antifibrotic effects [52-54]. In the present study, the result showed that the effect of iNOS on egg-induced lung fibrosis was negatively correlated with the expression of TGF-β3, which suggests that TGF-β3 may protect against the development of pulmonary fibrosis. This idea confirms earlier studies showing that TGF-β3 decelerates the progress of radiation-induced pulmonary fibrosis by hindering fibrocyte recruitment and regulating the IFN-γ/IL-4 balance [53].

In conclusion, these results reveal that iNOS plays a critical role in the regulation of immune system reactivity, particularly in Th1/Th2-associated cytokine responses and chemokine production in rats, during *S. japonicum* infection, which leads to a rapid rejection of schistosome survival and no granuloma formation. Furthermore, we provide direct evidence that high levels of NO in rats can promote the development of fibrosis, induced by inflammation, and suggests a possible cause of rapid inflammatory repair in the lungs and liver of rats with schistosomiasis. The key interactions, reported in this study, are schematically represented in Figure 7. Thus, this study significantly enhances our understanding of the immunoregulatory effect of NO on defense and immunopathological responses in rats.

Materials and Methods

Ethics statement

All animal work was approved by the Laboratory Animal Use and Care Committee of Sun Yat-Sen University under license number 2012CB53000 and conformed to the Chinese National Institute of Health Guide for the Care and Use of Laboratory Animals.

Animals and parasite infections

Sprague Dawley (SD) rats were purchased from the Medical Experimental Animal Center of Guangdong Province. iNOS-KO rats were generated with the transcription activator-like effector nuclease technology (TALENs) [55]. These deficient rats are viable, fertile and do not display any obvious appearance or physical abnormalities. Animals were kept in ventilated cages with available food and water under specific-pathogen-free conditions in the Sun Yat-Sen University following the university policy. Six to eight week old male rats were used for the study. In the infection experiments, rats and rabbits were percutaneously infected with 200 or 1000 cercariae of a Chinese mainland strain of *S. japonicum* obtained from infected *Oncomelania hupensis* snails, which were purchased from the Shanghai Institute of Parasitic Diseases (Shanghai, China). Parasites were perfused from the portal tract at 1, 4- and 7-weeks post-infection and kept in 0.9% saline. The collected worms were counted and photographed under a stereoscopic microscope (M205FA, Leica, Germany). For the induction of pulmonary granulomas, *S. japonicum* eggs were isolated and purified from the livers of infected rabbits. 15,000 eggs were delivered to the lungs of WT and iNOS-KO rats via tail vein injection (i.v.) [10, 28]. Animals were sacrificed on days 7 and 14 post-challenge, and the left lung was removed for fibrosis analysis.

Histopathology and fibrosis

Pulmonary and liver tissues were fixed in 4% neutral buffered formalin, embedded in paraffin for sectioning, dewaxed, and stained with Masson's trichrome for fibrosis analysis. Collagen is shown as blue in Masson staining, and the blue area reflects the amount of collagen. The severity of fibrosis was determined by the area of collagen using Image-Pro Plus software. All sections were imaged using an automatic slide scanning system (AxioScan.Z1, Zeiss, Germany) at 10X magnification.

Immunohistochemistry

For immunohistochemical analysis, the dewaxed sections were boiled in 10 mmol/L citrate buffer for 20 min for epitope retrieval following washing three times in PBS. After incubating in 3% hydrogen peroxide, the sections were blocked with 1% BSA for 1 hour. The slices were incubated with anti-rat antibodies against IL-10 (1:200, Servicebio, China), TGF-β3(1:200, Servicebio, China), IL-6 (1:400, Servicebio, China), TNF-α (1:300, Servicebio, China), IFN-γ (1:150, Servicebio, China) and α-SMA (1:1000, Servicebio, China) at 4ºC overnight and then incubated with HRP labelled Goat anti-rabbit IgG general secondary antibody (DAKO, Denmark). Finally, the antibody binding was visualized by using a DAB kit (Thermo Scientific). All sections were imaged using an automatic slide scanning system (AxioScan.Z1, Zeiss, Germany) at 20X magnification.

Flow cytometry

Spleens, mesenteric lymph node (LN), and livers from WT and iNOS-KO rats were removed aseptically and dispersed through a 70-um nylon strainer. Single-cell suspensions of peripheral blood, spleens, LN, and livers were prepared by Ficoll-Hypaque (Hao Yang Biological Manufacture, Tianjin, China) gradient centrifugation, washed twice, and suspended in PBS containing 0.1% BSA and 0.05% sodium azide. For surface staining, 2×10^6 cells per 100ul were incubated with CD3-APC (eBioscience, CA), CD19-FITC (Abcam, UK), CD4-PerCP-eFluor710 (eBioscience, CA), CD8-PE-Cy7 (eBioscience, CA), and macrophage marker-PE (eBioscience, CA) for 30 min at 4℃ in the dark. For the detection of intracellular cytokines, cells in complete RPMI 1640 medium were stimulated with PMA (20 ng/ml), Ionomycin (1 μg/ml), and Brefeldin A (10 μg/ml) (Sigma-Aldrich, Germany) for 6 h. Surface molecule staining CD3-FITC (BD Biosciences, USA), CD4-PE-Cy7 (eBioscience,

CA), and CD8-PerCP (eBioscience, CA) for 30min at 4℃ was carried out. Cells were fixed in 0.4% paraformaldehyde for 20 min, and permeabilized with 0.1% saponin buffer (Sigma-Aldrich, Germany), and stained for IFN-γ-APC (BD Biosciences, USA) and IL-4-PE (BD Biosciences, USA). The expression of surface molecules and intracellular molecules were analyzed on a BD LSR II flow cytometer using FlowJo v.8 software (Treestar, CA).

Cytokine analysis

The cytokines in serum samples were assayed using a rat 14plex cytokine kit (eBioscience, CA) according to the manufacturer's instructions. Samples were read using a Bio-Plex Suspension Array System (Biorad, USA).

RNA isolation and Real-time PCR

Liver tissues were placed in a 500ul Trizol reagent (Invitrogen, USA) and mashed with TissueLyser II (Qiagen, USA). Total RNA was isolated and further purified using RNeasy Mini Kit (Qiagen, USA) following the manufacturer's instructions. The concentration of RNA was quantified using a NanoDrop ND-1000 spectrophotometer (Nanodrop, USA). First-strand cDNA was synthesized using isolated RNA, Superscript II reverse transcriptase (Invitrogen, USA), and oligo dT as a primer. Genes expression were analyzed by quantitative real-time PCR (qRT-PCR) using the LightCycler480 real-time PCR system (Roche, Switzerland) and SYBR green qPCR Master Mixes (Roche, USA). The relative expression of each gene was normalized to the mean values for β-Actin before statistical analysis. β-Actin primers were: forward 5'- TGGAATCCTGTGGCATCCATGAAAC-3', reverse 5'-TAAAACGCAGCTCAGTAACAGTCCG-3'. IL-4 primers were: forward 5'-CACCCTGTTCTGCTTTCTCA-3', reverse 5'- CTCAGAGGGCTGTCGTTACA-3'.

IFN-γ primers were: forward 5’- AGGCCATCAGCAACAACATA-3’, reverse 5’- AGCTTTGTGCTGGATCTGTG-3’. IFN-γ primers were: forward 5’- AGGCCATCAGCAACAACATA-3’, reverse 5’- AGCTTTGTGCTGGATCTGTG-3’. IL-10 primers were: forward 5’- CCCAGAAATCAAGGAGCATT-3’, reverse 5’- TCATTCTTCACCTGCTCCAC-3’. TGF-β1 primers were: forward 5’- CTTGCCCTCTACAACCAACA-3’, reverse 5’- CTTGCGACCCACGTAGTAGA-3’. IL-6 primers were: forward 5’- ACTTCACAAGTCGGAGGCTT-3’, reverse 5’- AGTGCATCATCGCTGTTCAT-3’. TNF-α primers were: forward 5’- TAGCAAACCACCAAGCAGAG-3’, reverse 5’- CCACCAGTTGGTTGTCTTTG-3’. MCP-1 primers were: forward 5’- AATGAGTCGGCTGGAGAACT-3’, reverse 5’- CTGGACCCATTCCTTATTGG-3’. Eotaxin primers were: forward 5’- CAGCTCTCCACAGCACTTCT-3’, reverse 5’- GTCATGGTAAAGCAGCAGGA-3’. Eotaxin-2 primers were: forward 5’- CTCCACCACCATCATTGCTA -3’, reverse 5’- GAAATAAAGGTCACGCAGCA -3’. CXCL1 primers were: forward 5’- CACCCAAACCGAAGTCATAG-3’, reverse 5’-TTCAGGGTCAAGGCAAGC-3’. CXCL2 primers were: forward 5’- CGCCCAGACAGAAGTCATAG -3’, reverse 5’- GACGATCCTCTGAACCAAGG -3’. Relative expression was calculated using the $2^{-\Delta\Delta CT}$ method.

Scanning Electron Microscopy.

Parasites collected from infected mice, WT, and iNOS-KO rats at 7-day post-infection were fixed individually with 0.2M PBS containing 2.5% glutaraldehyde (pH 7.4) at 4 °C for 24 h. After washing three times with PBS and six times with distilled water, the samples were dehydrated in gradient ethanol and ethanol exchanged with acetone and isoamyl acetate. The tegument of the schistosomula was observed and photographed using a scanning electron microscope (S-2500, Hitachi) following critical point-drying and being coated with gold.

Statistical analysis

All statistical analyses were performed using SPSS 19 software. Significant differences between the two groups were determined using a student's unpaired T-test with Welch's correction or one-way ANOVA. Pearson correlation was used for correlation analysis. Graphs and analyses were performed using GraphPad Prism version 7.00 for Windows, San Diego, CA, USA. All data shown are presented as the mean ± SEM, and P values ≤0.05 were considered statistically significant.

Data Availability Statement: All relevant data are within the manuscript and its supporting information files.

Funding: This work was supported by grants from the National Natural Science Foundation of China (Grant No. 81802036 and 81871682), the National Key R&D Program of China (No. 2020YFC1200100 and No. 2016YFC1200500), the Natural Science Foundation of Guangdong Province of China (2020A1515010896), the China Postdoctoral Science Foundation (No. 2018M631027 and 2019T120770).

Competing interests: The authors have declared that no competing interests exist.

Acknowledgments

We would like to thank Juan Shen for assistance with the flow cytometry experiments and Ya-qiong Wang for scanning electron microscope (SEM) imaging.

Author contributions

Conceptualization: Zhao-Rong Lun, Zhong-Dao Wu, Jia Shen.

Data curation: Jia Shen, Si-fei Yu, Mei Peng.

Formal analysis: Jia Shen, Mei Peng.

Funding acquisition: Zhong-Dao Wu, Jia Shen.

Investigation: Jia Shen.

Methodology: Jia Shen, Si-fei Yu, Mei Peng, De-Hua Lai, Geoff Hide.

Project administration: Zhao-Rong Lun, Zhong-Dao Wu.

Resources: De-Hua Lai, Zhao-Rong Lun.

Writing – original draft: Jia Shen.

Writing – review & editing: all co-authors.

References

1. WHO. Schistosomiasis https://www.who.int/news-room/fact-sheets/detail/schistosomiasis 2021 [cited 2021 Jan 6].

2. Colley DG, Secor WE. Immunology of human schistosomiasis. Parasite Immunol. 2014; 36(8): 347-57. Epub 2014/08/22. doi: 10.1111/pim.12087. PubMed PMID: 25142505; PubMed Central PMCID: PMCPMC4278558.

3. He YX, Salafsky B, Ramaswamy K. Host--parasite relationships of *Schistosoma japonicum* in mammalian hosts. Trends Parasitol. 2001; 17(7): 320-4. Epub 2001/06/26. doi: 10.1016/s1471-4922(01)01904-3. PubMed PMID: 11423374.

4. Khalife J, Cetre C, Pierrot C, Capron M. Mechanisms of resistance to *S. mansoni* infection: the rat model. Parasitol Int. 2000; 49(4): 339-45. Epub 2000/11/15. doi: 10.1016/s1383-5769(00)00059-3. PubMed PMID: 11077269.

5. Capron M, Capron A. Rats, mice and men - models for immune effector mechanisms against schistosomiasis. Parasitol Today. 1986; 2(3): 69-75. Epub 1986/03/01. doi: 10.1016/0169-4758(86)90158-4. PubMed PMID: 15462774.

6. Caldas IR, Campi-Azevedo AC, Oliveira LF, Silveira AM, Oliveira RC, Gazzinelli G. Human *schistosomiasis mansoni*: immune responses during acute and chronic phases of the infection. Acta Trop. 2008; 108(2-3): 109-17. Epub 2008/06/26. doi: 10.1016/j.actatropica.2008.05.027. PubMed PMID: 18577364.
7. Lee CM, Elhelu MA, Aboko-Cole GF. Immunobiology of schistosomiasis. J Natl Med Assoc. 1979; 71(12): 1191-3. Epub 1979/12/01. PubMed PMID: 118264; PubMed Central PMCID: PMCPMC2537454.
8. Brunet LR, Dunne DW, Pearce EJ. Cytokine interaction and immune responses during *Schistosoma mansoni* infection. Parasitol Today. 1998; 14(10): 422-7. Epub 2006/10/17. doi: 10.1016/s0169-4758(98)01317-9. PubMed PMID: 17040834.
9. Cetre C, Pierrot C, Cocude C, Lafitte S, Capron A, Capron M, et al. Profiles of Th1 and Th2 cytokines after primary and secondary infection by *Schistosoma mansoni* in the semipermissive rat host. Infect Immun. 1999; 67(6): 2713-9. Epub 1999/05/25. PubMed PMID: 10338473; PubMed Central PMCID: PMCPMC96574.
10. Shen J, Lai DH, Wilson RA, Chen YF, Wang LF, Yu ZL, et al. Nitric oxide blocks the development of the human parasite *Schistosoma japonicum*. Proc Natl Acad Sci USA. 2017; 114(38): 10214-9. doi: 10.1073/pnas.1708578114. PubMed PMID: WOS:000411157100074.
11. Lowenstein CJ, Dinerman JL, Snyder SH. Nitric oxide: a physiologic messenger. Ann Intern Med. 1994; 120(3): 227-37. Epub 1994/02/01. doi: 10.7326/0003-4819-120-3-199402010-00009. PubMed PMID: 8273987.
12. Bogdan C. Nitric oxide synthase in innate and adaptive immunity: an update. Trends Immunol. 2015; 36(3): 161-78. Epub 2015/02/18. doi: 10.1016/j.it.2015.01.003. PubMed PMID: 25687683.
13. Tripathi P, Tripathi P, Kashyap L, Singh V. The role of nitric oxide in inflammatory reactions. FEMS Immunol Med Microbiol. 2007; 51(3): 443-52. Epub 2007/10/02. doi: 10.1111/j.1574-695X.2007.00329.x. PubMed PMID: 17903207.
14. Li Z, Zhao ZJ, Zhu XQ, Ren QS, Nie FF, Gao JM, et al. Differences in iNOS and

arginase expression and activity in the macrophages of rats are responsible for the resistance against *T. gondii* infection. PLoS ONE. 2012; 7(4): e35834. Epub 2012/05/05. doi: 10.1371/journal.pone.0035834. PubMed PMID: 22558235; PubMed Central PMCID: PMCPMC3338469.

15. Zhao ZJ, Zhang J, Wei J, Li Z, Wang T, Yi SQ, et al. Lower expression of inducible nitric oxide synthase and higher expression of arginase in rat alveolar macrophages are linked to their susceptibility to *Toxoplasma gondii* infection. PLoS ONE. 2013; 8(5): e63650. Epub 2013/05/22. doi: 10.1371/journal.pone.0063650. PubMed PMID: 23691079; PubMed Central PMCID: PMCPMC3655142.

16. Oswald IP, James SL. Nitrogen oxide in host defense against parasites. Methods. 1996; 10(1): 8-14. Epub 1996/08/01. doi: 10.1006/meth.1996.0072. PubMed PMID: 8812639.

17. Xue Q, Yan Y, Zhang R, Xiong H. Regulation of iNOS on immune cells and its role in diseases. Int J Mol Sci. 2018; 19(12): 3805. Epub 2018/12/07. doi: 10.3390/ijms19123805. PubMed PMID: 30501075; PubMed Central PMCID: PMCPMC6320759.

18. Liew FY. Regulation of lymphocyte functions by nitric oxide. Curr Opin Immunol. 1995; 7(3): 396-9. Epub 1995/06/01. doi: 10.1016/0952-7915(95)80116-2. PubMed PMID: 7546406.

19. Bogdan C. Nitric oxide and the immune response. Nat Immunol. 2001; 2(10): 907-16. Epub 2001/09/29. doi: 10.1038/ni1001-907. PubMed PMID: 11577346.

20. Taylor-Robinson AW. Counter-regulation of T helper 1 cell proliferation by nitric oxide and interleukin-2. Biochem Biophys Res Commun. 1997; 233(1): 14-9. Epub 1997/04/07. doi: 10.1006/bbrc.1997.6386. PubMed PMID: 9144387.

21. Niedbala W, Wei XQ, Piedrafita D, Xu D, Liew FY. Effects of nitric oxide on the induction and differentiation of Th1 cells. Eur J Immunol. 1999; 29(8): 2498-505. Epub 1999/08/24. doi: 10.1002/(SICI)1521-4141(199908)29:08<2498::AID-IMMU2498>3.0.CO;2-M. PubMed PMID: 10458764.

22. Bauer H, Jung T, Tsikas D, Stichtenoth DO, Frolich JC, Neumann C. Nitric oxide inhibits the secretion of T-helper 1- and T-helper 2-associated cytokines in activated human T cells. Immunology. 1997; 90(2): 205-11. Epub 1997/02/01. doi: 10.1046/j.1365-2567.1997.00161.x. PubMed PMID: 9135548; PubMed Central PMCID: PMCPMC1456757.
23. Beckman JS. Understanding peroxynitrite biochemistry and its potential for treating human diseases. Arch Biochem Biophys. 2009; 484(2): 114-6. Epub 2009/04/28. doi: 10.1016/j.abb.2009.03.013. PubMed PMID: 19393377; PubMed Central PMCID: PMCPMC2709800.
24. Wei XQ, Charles IG, Smith A, Ure J, Feng GJ, Huang FP, et al. Altered immune responses in mice lacking inducible nitric oxide synthase. Nature. 1995; 375(6530): 408-11. Epub 1995/06/01. doi: 10.1038/375408a0. PubMed PMID: 7539113.
25. Gajewski TF, Schell SR, Fitch FW. Evidence implicating utilization of different T cell receptor-associated signaling pathways by TH1 and TH2 clones. J Immunol. 1990; 144(11): 4110-20. Epub 1990/06/01. PubMed PMID: 2140386.
26. Huang FP, Niedbala W, Wei XQ, Xu D, Feng GJ, Robinson JH, et al. Nitric oxide regulates Th1 cell development through the inhibition of IL-12 synthesis by macrophages. Eur J Immunol. 1998; 28(12): 4062-70. Epub 1998/12/23. doi: 10.1002/(SICI)1521-4141(199812)28:12<4062::AID-IMMU4062>3.0.CO;2-K. PubMed PMID: 9862342.
27. Ihrig M, Whary MT, Dangler CA, Fox JG. Gastric helicobacter infection induces a Th2 phenotype but does not elevate serum cholesterol in mice lacking inducible nitric oxide synthase. Infect Immun. 2005; 73(3): 1664-70. Epub 2005/02/26. doi: 10.1128/IAI.73.3.1664-1670.2005. PubMed PMID: 15731067; PubMed Central PMCID: PMCPMC1064950.
28. Wynn TA, Eltoum I, Cheever AW, Lewis FA, Gause WC, Sher A. Analysis of cytokine mRNA expression during primary granuloma formation induced by eggs of *Schistosoma mansoni*. J Immunol. 1993; 151(3): 1430-40. Epub 1993/08/01. PubMed

PMID: 8335939.
29. Wynn TA, Eltoum I, Oswald IP, Cheever AW, Sher A. Endogenous interleukin 12 (IL-12) regulates granuloma formation induced by eggs of *Schistosoma mansoni* and exogenous IL-12 both inhibits and prophylactically immunizes against egg pathology. J Exp Med. 1994; 179(5): 1551-61. Epub 1994/05/01. doi: 10.1084/jem.179.5.1551. PubMed PMID: 7909326; PubMed Central PMCID: PMCPMC2191492.
30. Pearce EJ, MacDonald AS. The immunobiology of schistosomiasis. Nat Rev Immunol. 2002; 2(7): 499-511. Epub 2002/07/03. doi: 10.1038/nri843. PubMed PMID: 12094224.
31. Coulson PS, Smythies LE, Betts C, Mabbott NA, Sternberg JM, Wei XG, et al. Nitric oxide produced in the lungs of mice immunized with the radiation-attenuated schistosome vaccine is not the major agent causing challenge parasite elimination. Immunology. 1998; 93(1): 55-63. Epub 1998/04/16. doi: 10.1046/j.1365-2567.1998.00405.x. PubMed PMID: 9536119; PubMed Central PMCID: PMCPMC1364106.
32. Yixun He, Peng X. Scanning electron microscopy of *Schistosorna japonicum* during growth and maturation in mouse. Acta Zoologica Sinica. 1985; 31(2):138-42.
33. Constant SL, Wilson RA. In vivo lymphocyte responses in the draining lymph nodes of mice exposed to *Schistosoma mansoni*: preferential proliferation of T cells is central to the induction of protective immunity. Cell Immunol. 1992; 139(1): 145-61. Epub 1992/01/01. doi: 10.1016/0008-8749(92)90108-2. PubMed PMID: 1728963.
34. Mountford AP, Coulson PS, Pemberton RM, Smythies LE, Wilson RA. The generation of interferon-gamma-producing T lymphocytes in skin-draining lymph nodes, and their recruitment to the lungs, is associated with protective immunity to *Schistosoma mansoni*. Immunology. 1992;7 5(2): 250-6. Epub 1992/02/01. PubMed PMID: 1532378; PubMed Central PMCID: PMCPMC1384702.
35. Ferru I, Roye O, Delacre M, Auriault C, Wolowczuk I. Infection of B-cell-deficient mice by the parasite *Schistosoma mansoni*: demonstration of the

participation of B cells in granuloma modulation. Scand J Immunol. 1998; 48(3): 233-40. Epub 1998/09/22. doi: 10.1046/j.1365-3083.1998.00376.x. PubMed PMID: 9743206.
36. Kalinkovich A, Weisman Z, Greenberg Z, Nahmias J, Eitan S, Stein M, et al. Decreased CD4 and increased CD8 counts with T cell activation is associated with chronic helminth infection. Clin Exp Immunol. 1998; 114(3): 414-21. Epub 1998/12/09. doi: 10.1046/j.1365-2249.1998.00736.x. PubMed PMID: 9844052; PubMed Central PMCID: PMCPMC1905129.
37. Shiku H. Importance of CD4+ helper T-cells in antitumor immunity. Int J Hematol. 2003; 77(5): 435-8. Epub 2003/07/05. doi: 10.1007/BF02986610. PubMed PMID: 12841380.
38. Bennett SR, Carbone FR, Karamalis F, Miller JF, Heath WR. Induction of a CD8+ cytotoxic T lymphocyte response by cross-priming requires cognate CD4+ T cell help. J Exp Med. 1997; 186(1): 65-70. Epub 1997/07/07. doi: 10.1084/jem.186.1.65. PubMed PMID: 9206998; PubMed Central PMCID: PMCPMC2198964.
39. Chensue SW, Warmington KS, Hershey SD, Terebuh PD, Othman M, Kunkel SL. Evolving T cell responses in murine schistosomiasis. Th2 cells mediate secondary granulomatous hypersensitivity and are regulated by CD8+ T cells in vivo. J Immunol. 1993; 151(3): 1391-400. Epub 1993/08/01. PubMed PMID: 8335935.
40. James SL, Cheever AW, Caspar P, Wynn TA. Inducible nitric oxide synthase-deficient mice develop enhanced type 1 cytokine-associated cellular and humoral immune responses after vaccination with attenuated *Schistosoma mansoni* cercariae but display partially reduced resistance. Infect Immun. 1998; 66(8): 3510-8. Epub 1998/07/23. PubMed PMID: 9673227; PubMed Central PMCID: PMCPMC108380.
41. Hesse M, Cheever AW, Jankovic D, Wynn TA. NOS-2 mediates the protective anti-inflammatory and antifibrotic effects of the Th1-inducing adjuvant, IL-12, in a Th2 model of granulomatous disease. Am J Pathol. 2000; 157(3): 945-55. Epub 2000/09/12. doi: 10.1016/S0002-9440(10)64607-X. PubMed PMID: 10980133;

PubMed Central PMCID: PMCPMC1885696.
42. McGovern KE, Wilson EH. Role of chemokines and trafficking of immune cells in parasitic infections. Curr Immunol Rev. 2013; 9(3): 157-68. Epub 2013/01/01. doi: 10.2174/1573395509666131217000000. PubMed PMID: 25383073; PubMed Central PMCID: PMCPMC4223871.
43. Hsu YC, Wang LF, Chien YW. Nitric oxide in the pathogenesis of diffuse pulmonary fibrosis. Free Radic Biol Med. 2007; 42(5): 599-607. Epub 2007/02/13. doi: 10.1016/j.freeradbiomed.2006.11.031. PubMed PMID: 17291983.
44. Anavi S, Eisenberg-Bord M, Hahn-Obercyger M, Genin O, Pines M, Tirosh O. The role of iNOS in cholesterol-induced liver fibrosis. Lab Invest. 2015; 95(8): 914-24. Epub 2015/06/23. doi: 10.1038/labinvest.2015.67. PubMed PMID: 26097999.
45. Genovese T, Cuzzocrea S, Di Paola R, Failla M, Mazzon E, Sortino MA, et al. Inhibition or knock out of inducible nitric oxide synthase result in resistance to bleomycin-induced lung injury. Respir Res. 2005; 6(1):58. Epub 2005/06/16. doi: 10.1186/1465-9921-6-58. PubMed PMID: 15955252; PubMed Central PMCID: PMCPMC1177992.
46. Noguchi S, Yatera K, Wang KY, Oda K, Akata K, Yamasaki K, et al. Nitric oxide exerts protective effects against bleomycin-induced pulmonary fibrosis in mice. Respir Res. 2014; 15(1):92. Epub 2014/08/06. doi: 10.1186/s12931-014-0092-3. PubMed PMID: 25092105; PubMed Central PMCID: PMCPMC4237963.
47. Naura AS, Zerfaoui M, Kim H, Abd Elmageed ZY, Rodriguez PC, Hans CP, et al. Requirement for inducible nitric oxide synthase in chronic allergen exposure-induced pulmonary fibrosis but not inflammation. J Immunol. 2010; 185(5): 3076-85. Epub 2010/07/30. doi: 10.4049/jimmunol.0904214. PubMed PMID: 20668217; PubMed Central PMCID: PMCPMC3077076.
48. Biernacka A, Dobaczewski M, Frangogiannis NG. TGF-beta signaling in fibrosis. Growth Factors. 2011; 29(5): 196-202. Epub 2011/07/12. doi: 10.3109/ 08977194. 2011.595714. PubMed PMID: 21740331; PubMed Central PMCID:

PMCPMC4408550.
49. Sureshbabu A, Muhsin SA, Choi ME. TGF-beta signaling in the kidney: profibrotic and protective effects. Am J Physiol Renal Physiol. 2016; 310(7): F596-F606. Epub 2016/01/08. doi: 10.1152/ajprenal.00365.2015. PubMed PMID: 26739888; PubMed Central PMCID: PMCPMC4824143.
50. Border WA, Noble NA. Transforming growth factor beta in tissue fibrosis. N Engl J Med. 1994; 331(19): 1286-92. Epub 1994/11/10. doi: 10.1056/ NEJM 199411103311907. PubMed PMID: 7935686.
51. Kinoshita K, Aono Y, Azuma M, Kishi J, Takezaki A, Kishi M, et al. Antifibrotic effects of focal adhesion kinase inhibitor in bleomycin-induced pulmonary fibrosis in mice. Am J Respir Cell Mol Biol. 2013; 49(4): 536-43. Epub 2013/05/07. doi: 10.1165/rcmb.2012-0277OC. PubMed PMID: 23642017.
52. Prelog M, Scheidegger P, Peter S, Gershwin ME, Wick G, Sgonc R. Diminished transforming growth factor beta2 production leads to increased expression of a profibrotic procollagen alpha2 type I messenger RNA variant in embryonic fibroblasts of UCD-200 chickens, a model for systemic sclerosis. Arthritis Rheum. 2005; 52(6): 1804-11. Epub 2005/06/04. doi: 10.1002/art.21109. PubMed PMID: 15934083.
53. Xu L, Xiong S, Guo R, Yang Z, Wang Q, Xiao F, et al. Transforming growth factor beta3 attenuates the development of radiation-induced pulmonary fibrosis in mice by decreasing fibrocyte recruitment and regulating IFN-gamma/IL-4 balance. Immunol Lett. 2014; 162(1 Pt A): 27-33. Epub 2014/07/06. doi: 10.1016/j.imlet. 2014.06.010. PubMed PMID: 24996042.
54. Occleston NL, O'Kane S, Laverty HG, Cooper M, Fairlamb D, Mason T, et al. Discovery and development of avotermin (recombinant human transforming growth factor beta 3): a new class of prophylactic therapeutic for the improvement of scarring. Wound Repair Regen. 2011; 19 Suppl 1:s38-48. Epub 2011/08/04. doi: 10.1111/j.1524-475X.2011.00711.x. PubMed PMID: 21793965.
55. Joung JK, Sander JD. TALENs: a widely applicable technology for targeted

genome editing. Nat Rev Mol Cell Biol. 2013; 14(1): 49-55. Epub 2012/11/22. doi: 10.1038/nrm3486. PubMed PMID: 23169466; PubMed Central PMCID: PMCPMC3547402.

Figure legends

Figure 1. iNOS-KO rats show slow clearance of *S. japonicum*, exacerbated pathological responses and increased morbidity. Wildtype (WT) and iNOS knock-out (KO) rats were infected with 200 *S. japonicum* cercariae, parasites were perfused from the portal tract at 1, 4- and 7-weeks post-infection and counted under a stereoscopic microscope. A. Percentage worm load collected from hepatic portal vein infusion. The data are expressed as the mean ± SEM of 5 rats per group. $^{*}P < 0.05$, $^{**}P < 0.01$. B. Micrographs of representative parasites from WT and iNOS-KO rats. ♂, male worms; ♀, female worms. Bar = 3.52mm. C. Histopathology of *S. japonicum* infected WT and iNOS-KO rats. Liver sections were stained with Masson's Trichrome at 7- and 12-weeks post-infection. Blue (aniline blue) represents collagen fibers. Red arrows indicate the granulomas, and the yellow arrow indicates the worm. Bar = 100 um. D. Weight change throughout 7 weeks period. n = 5 per group. The data are expressed as the mean ± SEM of 5 rats per group. $^{*}P < 0.05$, $^{**}P < 0.01$. E. Percentage survival of WT and iNOS-KO rats following a high dose (1000 cercariae) infection of *S. japonicum*. n = 9 per group.

Figure 2. Flow cytometry analysis of the changes in T cell and B cell proportions in iNOS-KO and WT control rats. WT and KO rats were infected percutaneously with 200 *S. japonicum* cercariae and sacrificed at 7 weeks post-infection. (A) Representative FACS plots of $CD3^{+}$ T cells and $CD19^{+}$ B cells in the spleens, LN, and livers. (B) Frequency of $CD3^{+}$ T cells and $CD19^{+}$ B cells in the spleens, LN, and livers. (C) Representative FACS plots of $CD4^{+}$ and $CD8^{+}$ T cells in the spleens, LN, and livers. Numbers represent the frequency of the boxed population within the $CD3^{+}$

T cell population. (D) Frequency of $CD4^+$ and $CD8^+$ T cells. (E) $CD4^+/CD8^+$ ratio. Data shown are means ± SEM and repeated twice with similar results, n = 5 rats per group. Significant differences have been noted, * P<0.05, $^{**}P$<0.01,$^{***}P$＜0.001. SP= Spleen, LN= lymph node.

Figure 3. A pro-inflammatory and pro-fibrogenic cytokine profile developed in infected iNOS-KO rats. WT and KO rats were infected percutaneously with 200 *S. japonicum* cercariae and serum was obtained for cytokine expression analysis by Multiplex at 0, 1, 4, 7, and 12-weeks post-infection. (A) Pro-inflammatory cytokines TNF-α, IL-1β, and IL-6 were significantly elevated in infected KO rats. (B) Th1 and Th2 cytokines were down-regulated in infected KO rats. (C) IL-2 and IL-13 were significantly up-regulated in infected KO rats. (D) The immunosuppressive cytokine IL-10 was significantly down-regulated in infected KO rats. n = 5 rats per group. Data shown are mean ± SEM and repeated twice with similar results. Significant differences have been noted, $^{*}P$<0.05, $^{**}P$<0.01,$^{***}P$ < 0.001.

Figure 4. iNOS deficiency impairs *S. japonicum*-elicited Th1 and Th2 responses in rats. Peripheral blood, mesenteric Lymph Nodes (LN) and splenic leukocytes were isolated from naive and 7-week *S. japonicum*-infected WT and KO rats. The they were stimulated with PMA/Ionomycin/BFA for 6 hours for analysis of the production of IFN-γ and IL-4 in $CD4^+$ T cells by flow cytometry. (A) Representative FACS plots of IFN-γ producing $CD4^+$ T cells in the blood, LN, and spleen. (B) Frequency of $CD4^+$ T cells producing IFN-γ. (C) Representative FACS plots of IL-4 producing $CD4^+$ T cells in the blood, LN, and spleen. (D) Frequency of $CD4^+$ T cells producing IL-4. Results for individual rats are shown and statistically significant differences are indicated. Data shown are mean ± SEM and repeated twice with similar results, n = 5 rats per group. $^{*}P$<0.05, $^{**}P$<0.01,$^{***}P$＜0.001.

Figure 5. Cytokine and chemokine production is impaired in the liver of infected iNOS-KO rats. WT and KO rats were infected percutaneously with 200 *S. japonicum*

cercariae and sacrificed at 7 weeks post-infection. (A) Relative mRNA levels of cytokines and chemokines in liver tissue were determined by real-time-PCR. Expression is normalized to β-actin. Data shown are mean ± SEM and repeated twice with similar results, n = 5 rats per group. Significant differences have been noted, $^{*}P < 0.05$, $^{**}P < 0.01$, $^{***}P < 0.001$. (B) Hepatic granuloma-associated cytokine expression was detected by immunohistochemistry (IHC). Brown (arrow) indicates a positive signal (Scale bars: 20 μm).

Figure 6. The progression of pulmonary granuloma-associated fibrosis is slowed in iNOS-KO rats. 15,000 eggs collected from *S. japonicum* infected rabbits were delivered to the lungs of WT and iNOS-KO rats via tail vein injection (i.v.), and lungs were removed for fibrosis analysis on 7 and 14 days post-challenge. (A) Representative images of pulmonary granuloma-associated fibrosis stained with Masson's Trichrome. Blue represents collagen fibers. (B) Fibrosis was quantified from Masson's Trichrome stained lung sections. (C) The immunohistochemistry results of α-SMA in lungs. Brown indicates the α-SMA signals. Scale bars=50 μm. (D) Quantitation of the expression of α-SMA. (E) Representative images of pulmonary granuloma associated cytokines expression by immunohistochemistry. Brown indicates the positive signals (Scale bars: 20 μm). E, egg. (F) Quantitation of the expression of cytokines. The data are expressed as the mean ± SEM of 4 rats per group. Significant differences have been noted, $^{*}P < 0.05$, $^{**}P < 0.01$. IOD, integrated optical density. (G) Correlation between the level of TGF-β3 and fibrosis from Masson's Trichrome was analyzed by Pearson correlation analysis (r = −0.55; *P*= 0.035). (D) Correlation between the level of TGF-β3 and α-SMA (r = −0.46; *P*= 0.082).

Figure 7. Schematic model for the role of iNOS in maintaining host immune activity against *S. japonicum* infection in rats. When iNOS is present in rats, T-cell immune responses were rapidly activated upon infection with *S. japonicum*, with associated consequences: $CD3^+$ T cells and $CD4^+$ T cells were depleted, the ratio of

$CD4^+$ / $CD8^+$ decreased, Th2 and Th1 immune cells upregulated, and the secretion of cytokines and chemokines increased. These immune effects mediated by iNOS lead to rapid elimination of schistosome, inhibition of worm development and no typical egg-granuloma formation in rats. Therefore, schistosomiasis does not cause death in rats. However, T cell responses were impaired in the absence of iNOS in rats, which resulted in delayed clearance of worms, increasing the development and oviposition of schistosomes, a lot of egg-granuloma formation which developed into fibrosis, and produced a lot of inflammatory cytokines, eventually leading to the death of the host.

Supporting information

S1 Fig. Flow cytometry analysis of the changes in macrophage proportions in iNOS-KO and WT control rats. WT and KO rats were infected percutaneously with 200 *S. japonicum* cercariae and sacrificed at 7 weeks post-infection. (A) Representative FACS plots of macrophages in the spleens, LN and livers. (B) Frequency of macrophages in the spleens, LN and livers. Results for individual rats are shown and statistically significant differences are indicated. Data shown are mean ± SEM and repeated twice with similar results. n = 5 rats per group. No significant differences are found. LN= lymph node.

S2 Fig. Scanning electron microscopy (SEM) analysis of the tegument the of schistosomula from BALB/c mouse, WT rat and iNOS-KO rat at 7 days post-infection. vs, ventral sucker; S, spine. Bar = 20 μm. The loss of spines in the middle part of the body of schistosomula was found in mice and iNOS-KO rats, while large numbers of spines on the tegument are still presented at the middle part of body of schistosomula obtained from WT rat, suggesting the parasites growth retardation.

Figure 1 Click here to access/download;Figure;Figure 1.tif

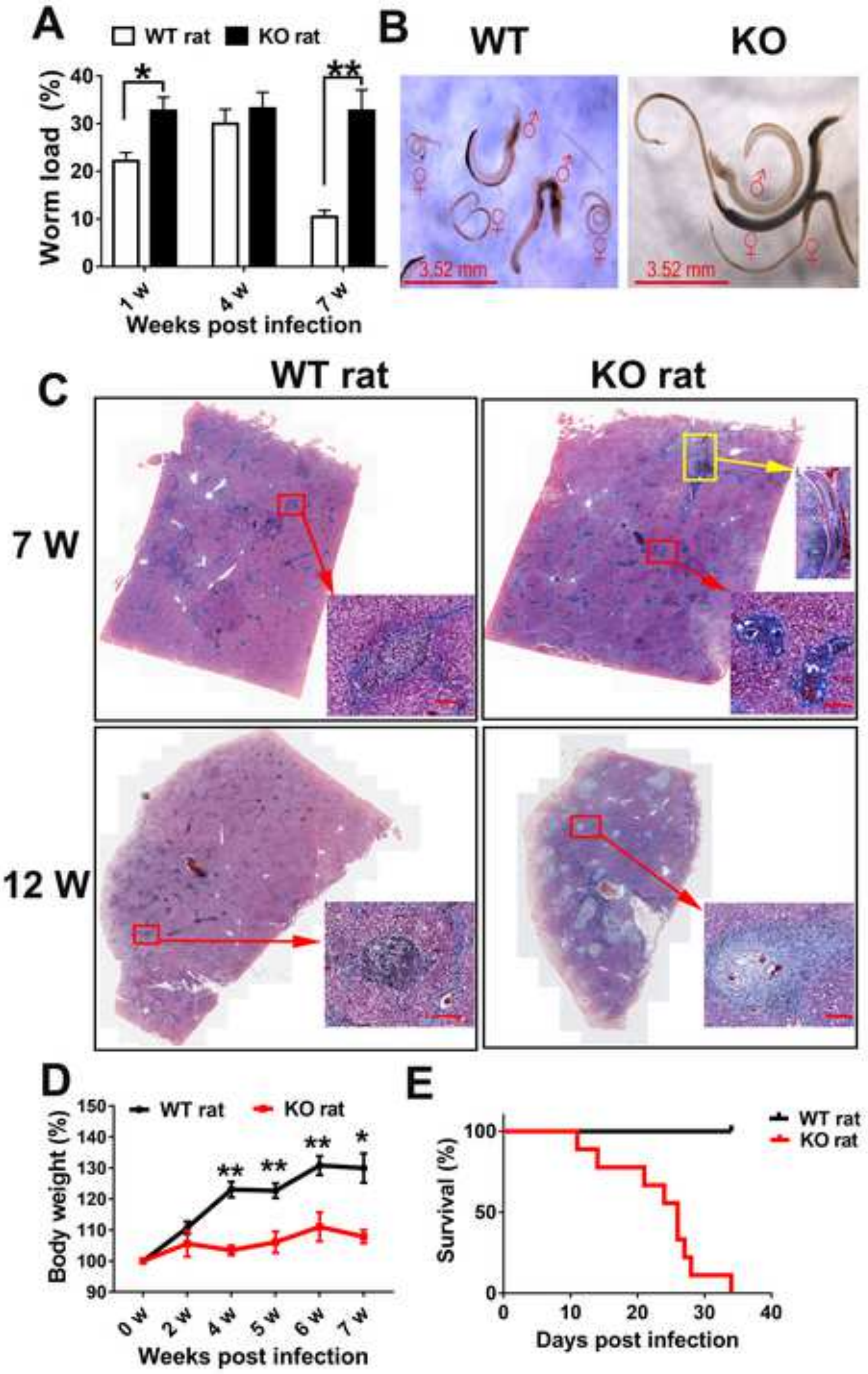

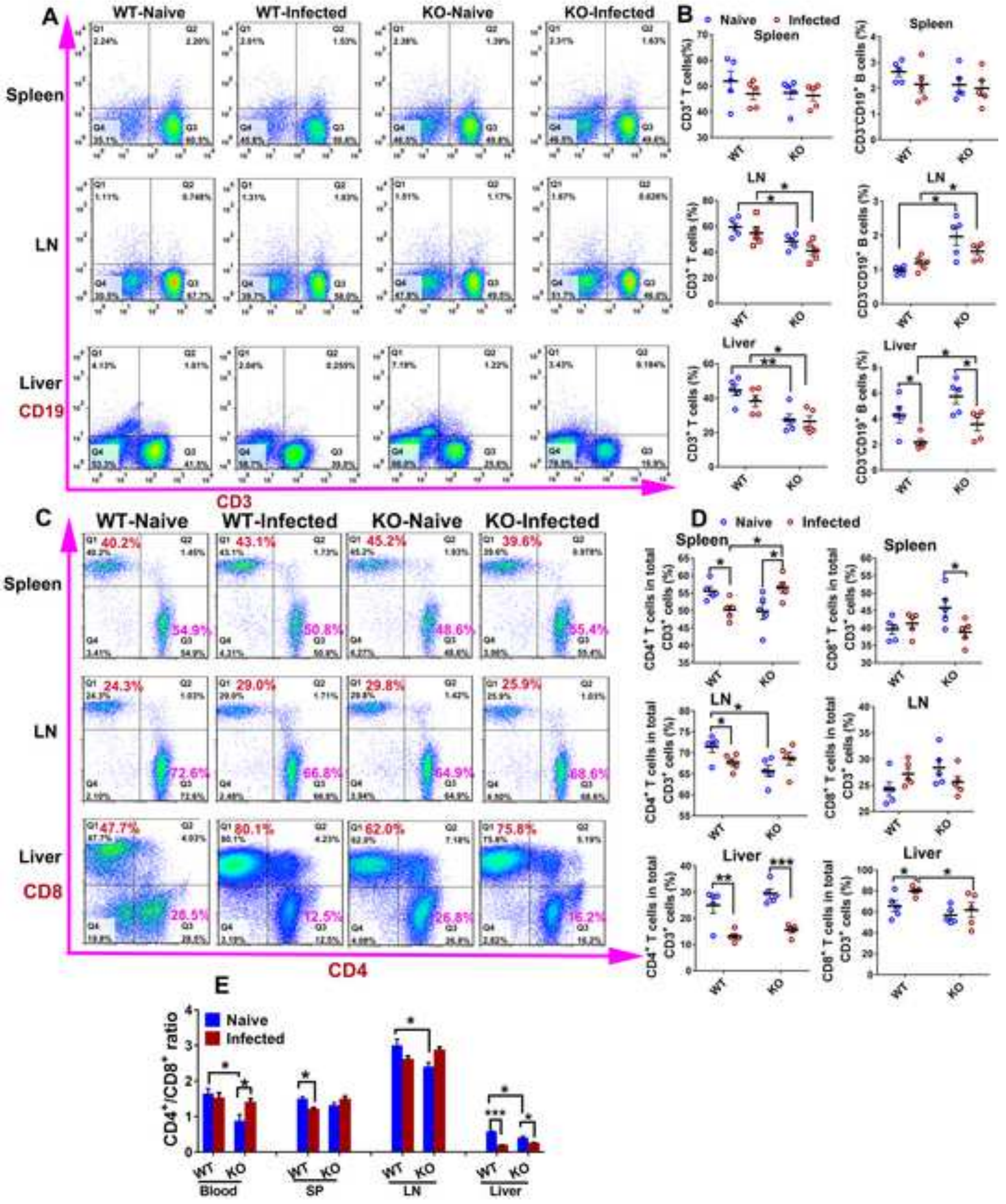
A
WT-Naive
WT-Infected
KO-Naive
KO-Infected
Spleen
LN
Liver
CD19
CD3
B
Naive
Infected
Spleen
LN
Liver
CD3+ T cells (%)
CD3-CD19+ B cells (%)
WT
KO
C
WT-Naive
WT-Infected
KO-Naive
KO-Infected
Spleen
LN
Liver
CD8
CD4
40.2%
43.1%
45.2%
39.6%
54.9%
50.8%
48.6%
55.4%
24.3%
29.0%
29.8%
25.9%
72.6%
66.8%
64.9%
68.6%
47.7%
80.1%
62.0%
75.8%
26.5%
12.5%
26.8%
16.2%
D
Naive
Infected
Spleen
LN
Liver
CD4+ T cells in total CD3+ cells (%)
CD8+ T cells in total CD3+ cells (%)
WT
KO
E
Naive
Infected
CD4+/CD8+ ratio
WT
KO
Blood
SP
LN
Liver

Figure 3

Click here to access/download;Figure;Figure 3.tif

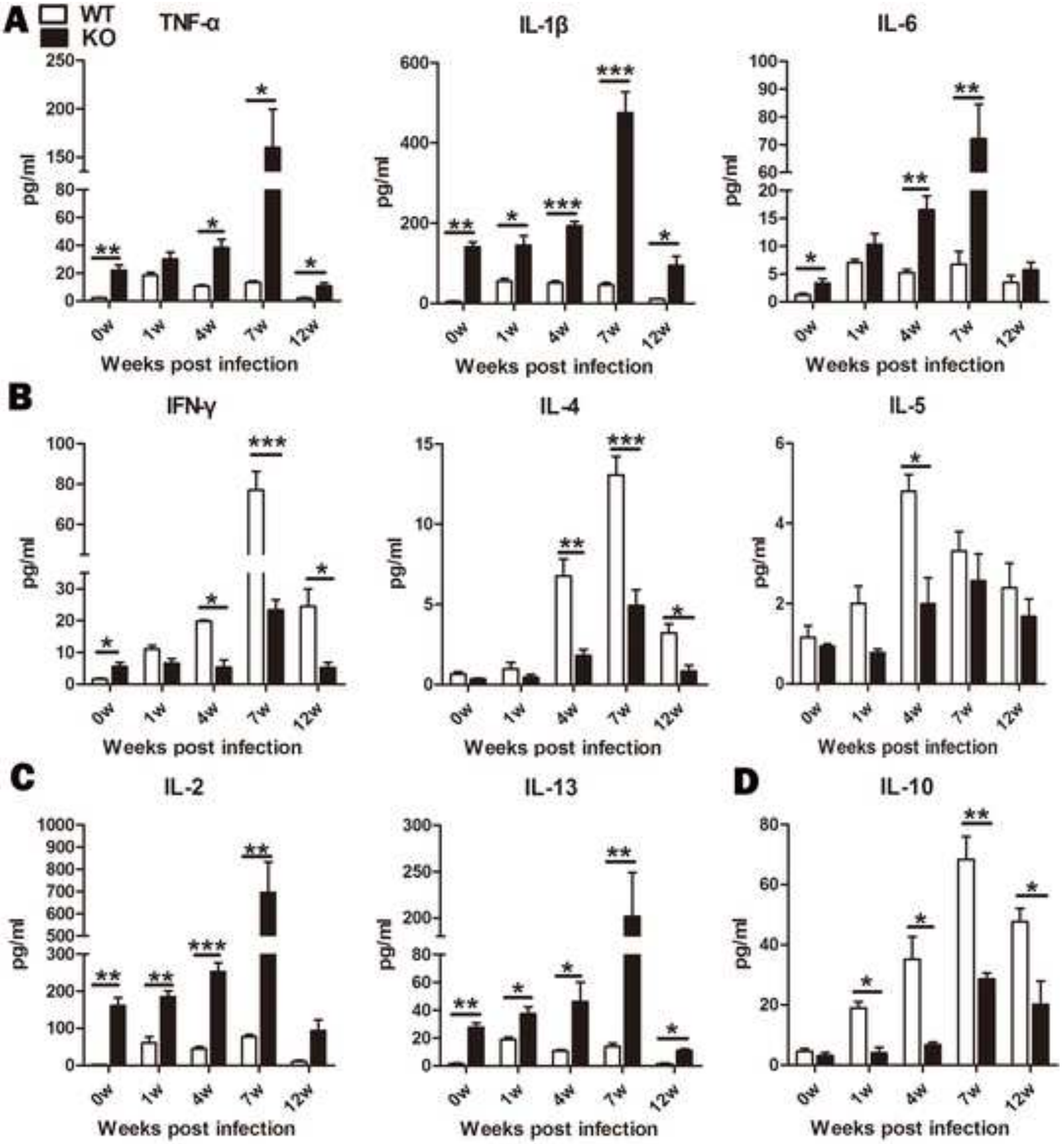

Figure 4

Click here to access/download;Figure;Figure 4.tif

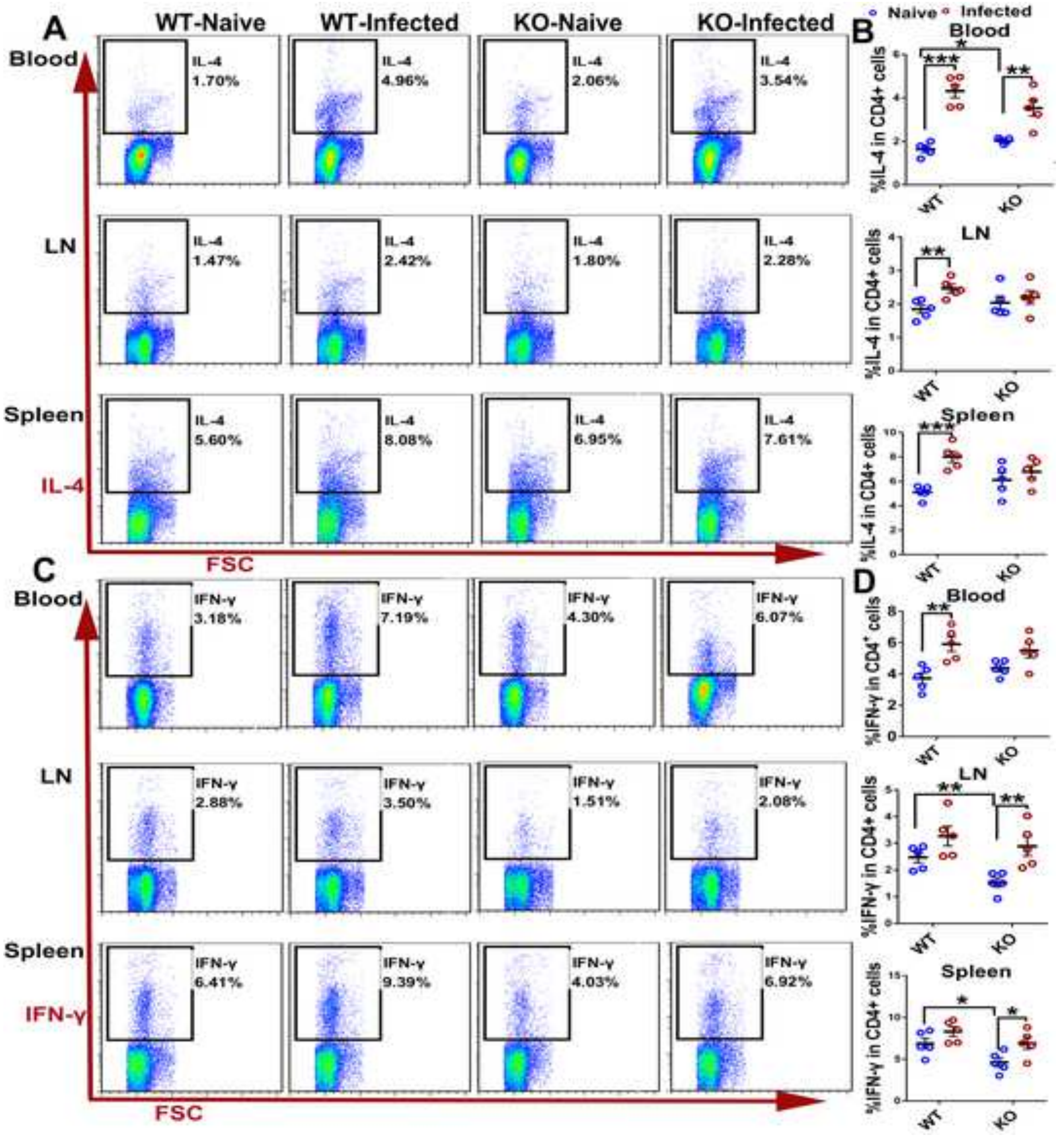

Figure 5

Click here to access/download;Figure;Figure 5.tif

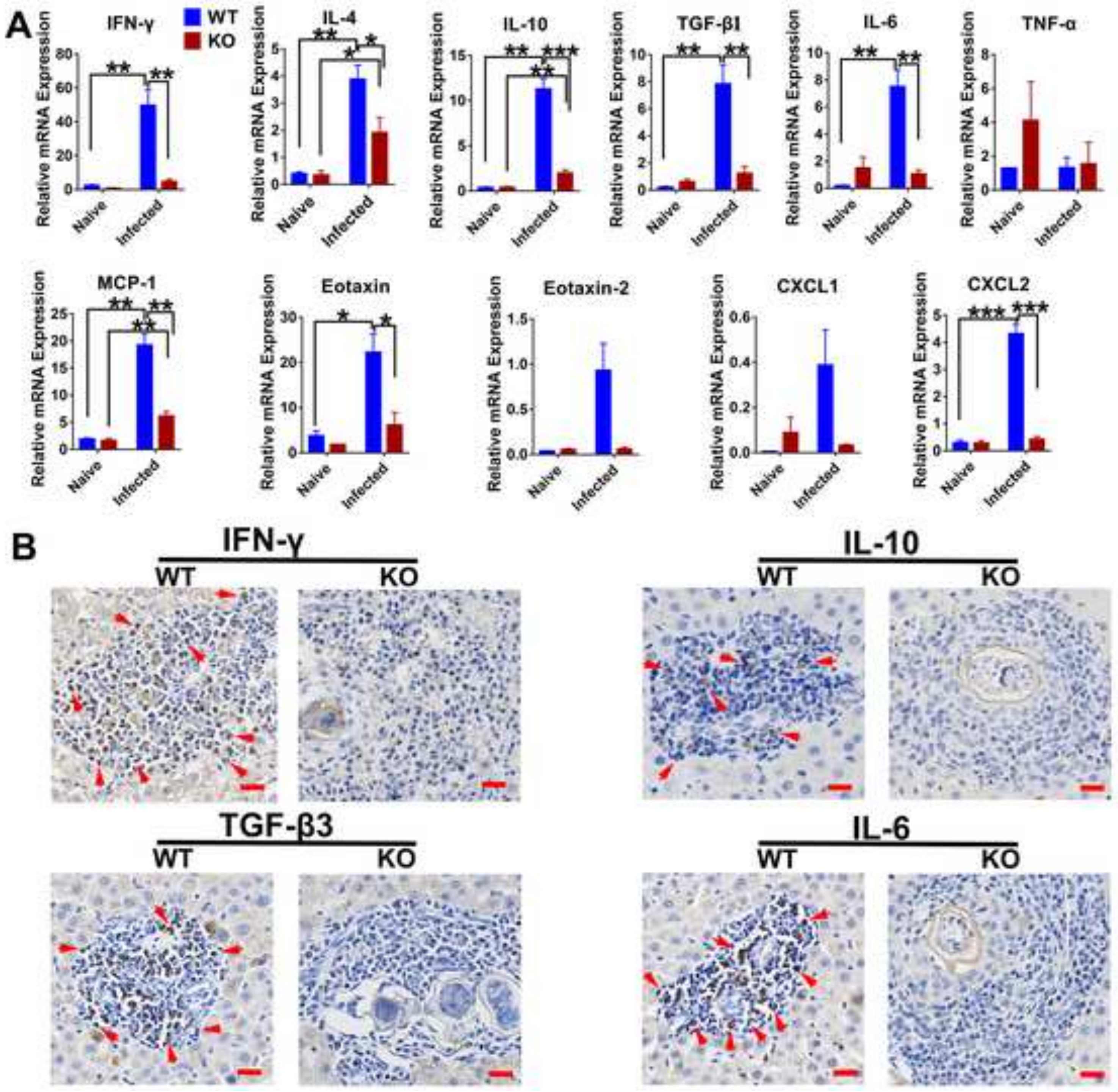

Figure 6

Click here to access/download;Figure;Figure 6.tif

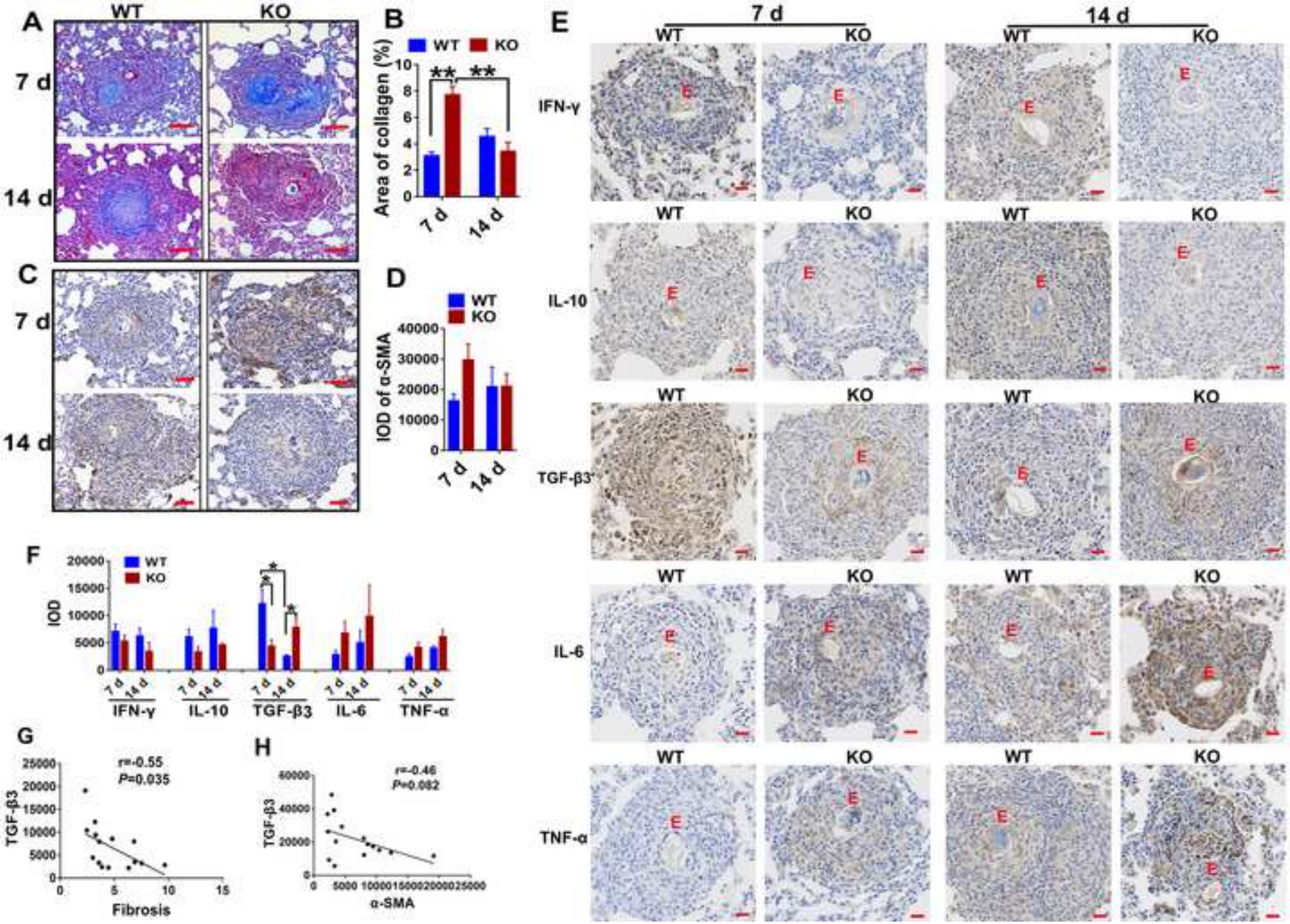

Figure 7

Click here to access/download;Figure;Figure 7.tif

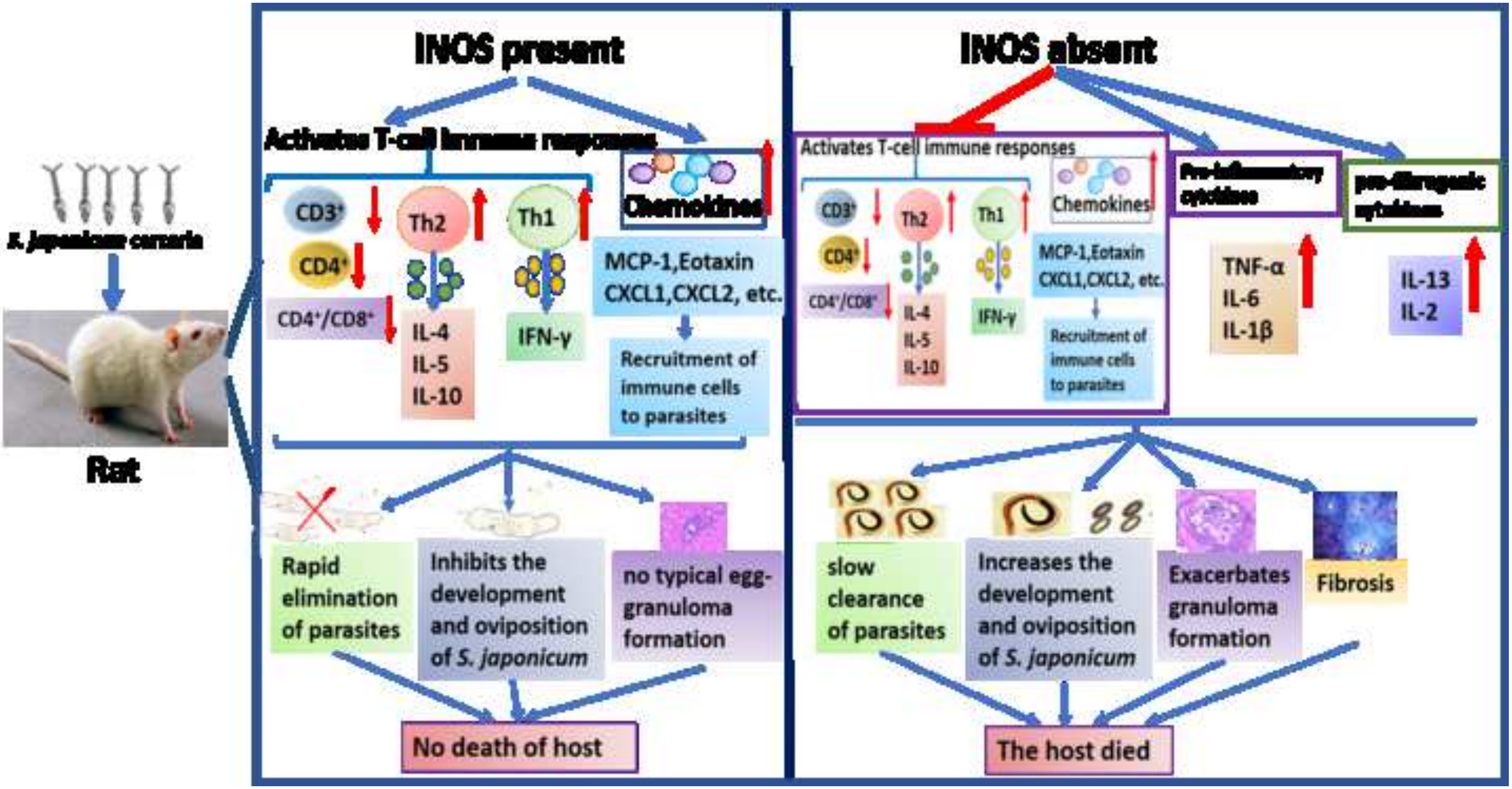

Fig. S1

Click here to access/download;Figure;Fig. S1.tif

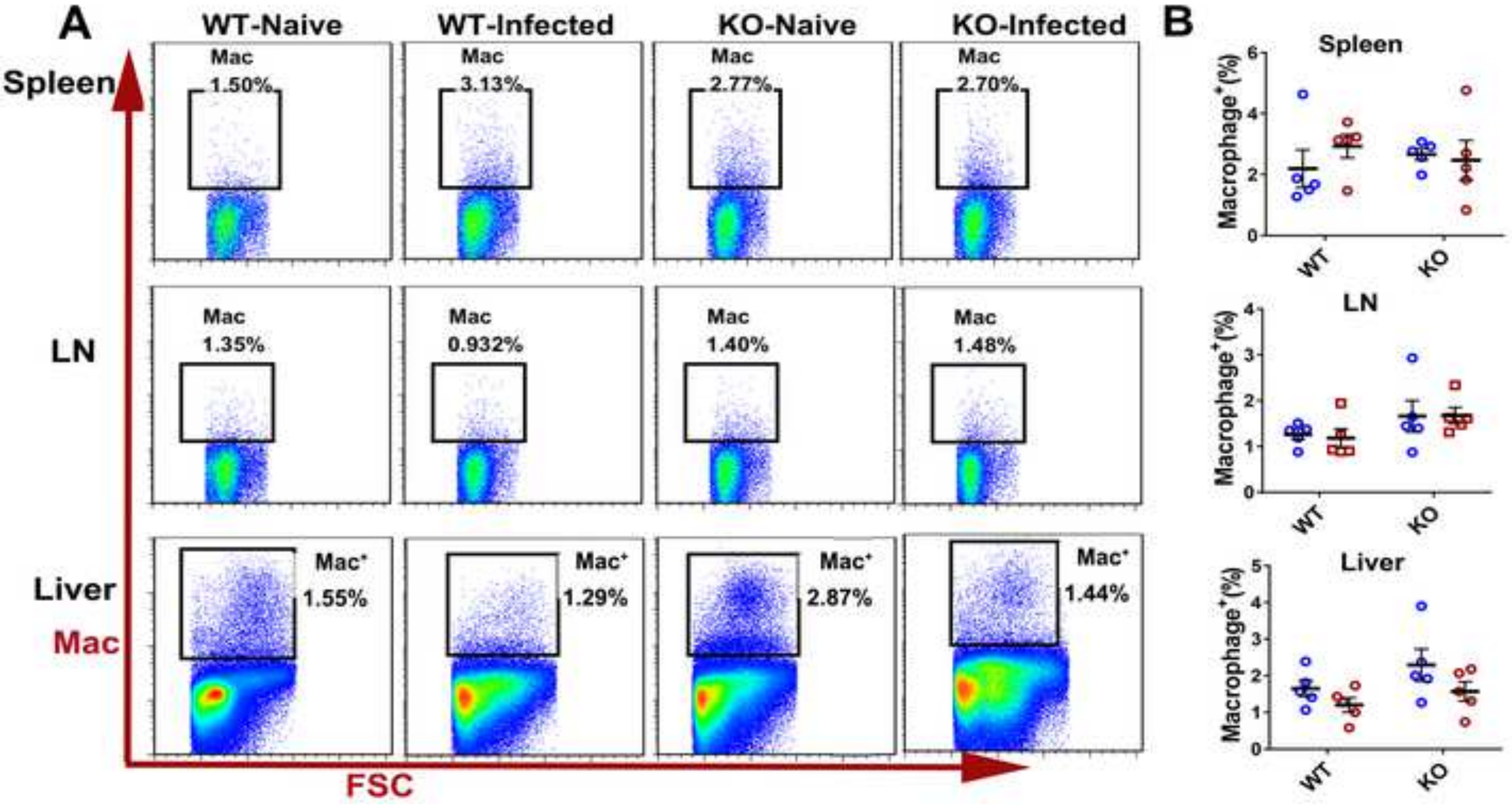

Fig. S2

Click here to access/download;Figure;Fig. S2.tif

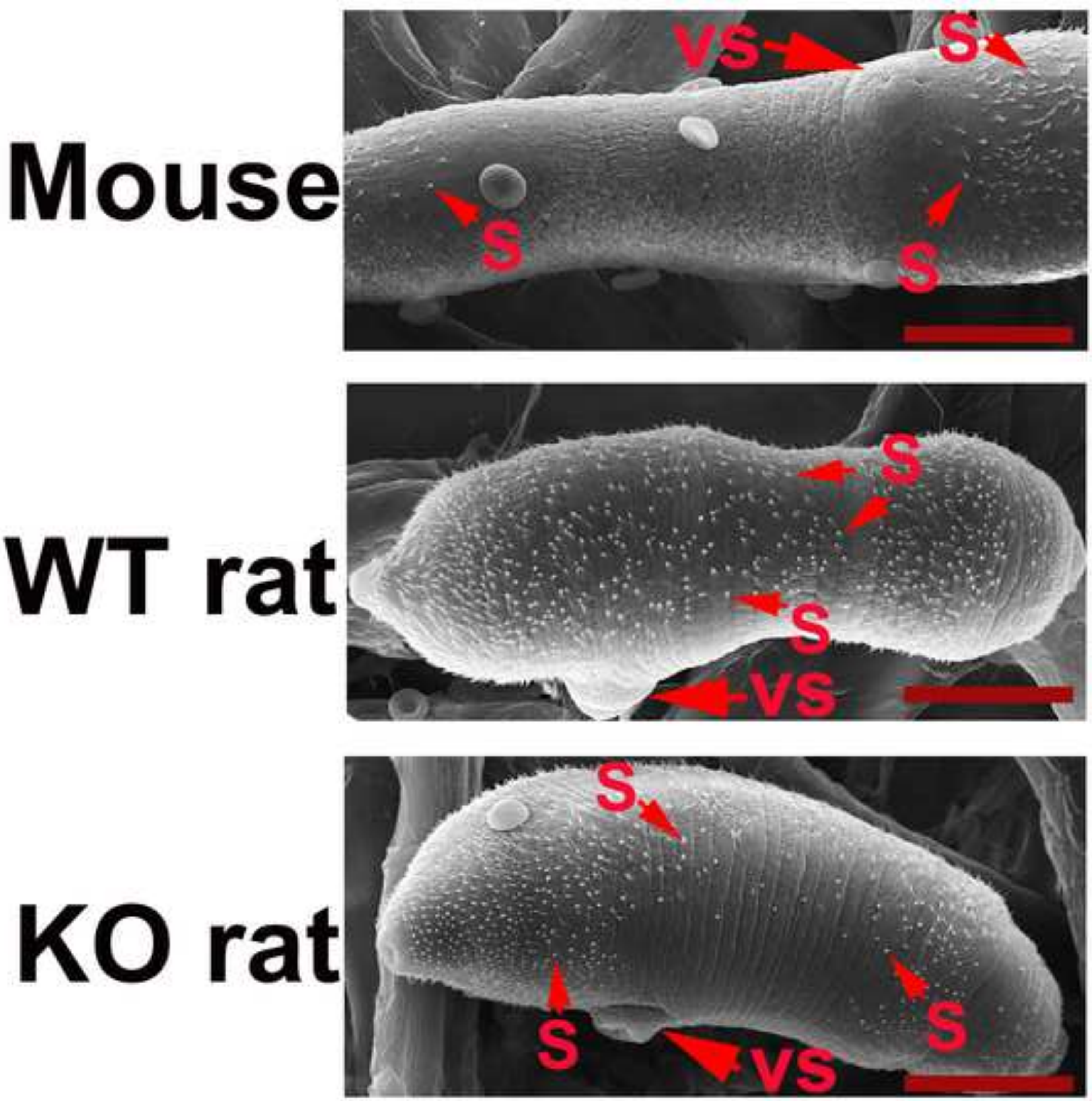

Supplement: Attachment — Submitted filename: PNTD-D-21-00912_reviewer.pdf [file pntd.0010403.s004.pdf]
