# Supplementary material for: iNOS is essential to maintain a protective Th1/Th2 response and the production of cytokines/chemokines against Schistosoma japonicum infection in rats
Source: PLoS Negl Trop Dis. 2022 May 18;16(5):e0010403. doi: 10.1371/journal.pntd.0010403 (PMC9116669; doi:10.1371/journal.pntd.0010403)
Supplement: Attachment — Submitted filename: The letter Response to Review comments.docx [file pntd.0010403.s005.docx]

Dear Reviewers,

Thank you for the review of our manuscript “iNOS is essential to maintain host immune activity against Schistosoma japonicum infection in rats”. We appreciate the constructive comments from you, which helped us to increase the clarity of the manuscript and interpret our data more accurately. We have tried our best to revise the manuscript. Please, see attached the point-by-point replies to the comments and the edited version of the manuscript with tracked changes.

We appreciate the efforts of you and hope that our re-submission will meet your expectations to be accepted for publication.

With the best wishes.

Yours sincerely,

Zhao-Rong Lun, PhD

On behalf of all co-authors

Professor in Zoology/Parasitology

State Key Laboratory of Biocontrol

Sun Yat-Sen University

Guangzhou 510275, P.R. China

Email: lsslzr@mail.sysu.edu.cn

**Methods**


-Are the objectives of the study clearly articulated with a clear testable hypothesis stated?

-Is the study design appropriate to address the stated objectives?

-Is the population clearly described and appropriate for the hypothesis being tested?

-Is the sample size sufficient to ensure adequate power to address the hypothesis being tested?

-Were correct statistical analysis used to support conclusions?

-Are there concerns about ethical or regulatory requirements being met?


Reviewer #1:

1.Can the authors specify the genetic background of iNOS-KO rats?

>>Thanks for the comment. We have supplemented the genetic background of iNOS-KO rats (Lines 612-613).

2.Please provide the Image-Pro Plus software version.

>>Thanks for the kind suggestion. In the revised manuscript, the Image-Pro Plus software version has been provided (Line 642).

3.Please provide the limit of quantitation of each assay and check whether all the data is within the range of quantification.

>> Thanks for the comment. We have added this content according to your advice (Line 683-688; 694-696).

4.Spacing errors in units (500 μL, 0.2 M, and so on). Please check it carefully.

>> Thanks again for your kind reminder. We have checked and corrected these errors.

5.In the section "RNA isolation and Real-time PCR", IFN-γ primers are duplicated, please check it carefully.
>> We are sorry for the carelessness and we have deleted the duplicate content (Line 713-714).

Reviewer #2: MAJOR CRITIQUES

The authors demonstrate that the iNOS-KO rat is a powerful model to investigate immunity to Schistosoma japonicum and shows no abnormal phenotype at baseline (pg 21). However in the absence of orthogonal lines of evidence, it is difficult to determine whether the experimental results are truly due to the direct involvement of iNOS and NO signaling—which is the authors’ central claim—or are confounded by secondary downstream effects of the iNOS knockout (such as abnormal early rat development). To provide the necessary evidence to support the central claim of their paper, the authors would need to perform the following new investigations:

- Rescue the iNOS knockout phenotype (partially or fully), via genetic re-introduction of an iNOS transgene into the knockout animals and/or via adoptive transfer of leukocytes (and particularly T cells) from WT rats into iNOS-KO rats.

- Use a non-genetic method, such as a small molecule iNOS inhibitor, to disrupt iNOS signaling in WT rats and demonstrate similar effects on S. japonicum and anti-parasite immune responses.

>> We would like to thank the reviewer very much for the critical comments and the suggestions. In fact, we have partially rescued the iNOS knockout phenotype via adoptive transfer of macrophages, which were considered to be the best-characterized source of NO as previously reported (*Annu Rev Immunol. 1997;15:323-50*), from WT rats into iNOS-KO rats. Our previously published results showed that the number of worms in the iNOS-KO rats with adoptive transfer were reduced and the development of *S. japonicum* was also partially inhibited (Shen J et al., PNAS, 2017,114:10214-19). The related results are shown as below:

Consistent with the above previously published results, the Th1 and Th2 immune responses in spleen, and the expression of cytokines and chemokines in liver, were increased in the iNOS-KO rats following adoptive transfer (Figure 6). We have provided these results in the revised manuscript as below:

Figure 6. Adoptive transfer of macrophages from WT rats into iNOS-KO rats partially restores the cytokine and chemokine production ability in infected KO rats. Adoptive transfer of wild type macrophages was performed in KO rats as described in Materials and Methods (KO + Mφ), and the infected KO rats that only received a PBS injection were used as the control group (KO). The rats were sacrificed at 6 weeks post-infection. (A) (B) IFN-γ and IL-4 concentration in supernatants of splenic leukocytes collected from infected KO and KO + Mφ rats after stimulated for 48 hours with SWA. (C-I) Hepatic granuloma-associated cytokine and chemokine expression were detected by immunohistochemistry. Brown (arrow) indicates a positive signal (Scale bars: 100 μm). The statistical results of the immunohistochemistry were shown as integrated optical density (IOD). Data are expressed as the mean ± SEM of 5 rats per group. Significant differences are indicated by ^*^*P* < 0.05, ^**^*P* < 0.01,^***^*P* ＜0.001.

The results are described as below:

“Our previous studies have shown that adoptive transfer of wild-type macrophages to KO rats, which could elevate the production of NO *in vivo*, could partially recover the resistance to *S. japonicum* in KO rats (*PNAS, 2017,114(38))*. Thus, to determine whether exogenous production of NO treatment could reverse the immune response profile of the KO rats, we separated the splenocytes from infected KO recipient rats (KO + Mφ) that received adoptive transfer macrophages and non-transferred controls, and examined the production of IFN-γ and IL-4. As expected, when adoptive transfer of wild-type macrophages was performed in KO rats, the production of IFN-γ and IL-4 in splenocytes was significantly increased compared with non-transferred controls (Fig. 6A and B).” (Lines 293-304).

“Similarly, the results were further confirmed by the rescue experiment using adoptive transfer of WT macrophages to KO rats, which partially restored cytokine and chemokine expression ability at the periphery of granulomas in the liver of infected KO rats (Fig. 6C-6I). (Lines 328-334).” We hope that the reviewer can accept our answer.

Reference:
Shen J, Lai DH, Wilson RA, Chen YF, Wang LF, Yu ZL, et al. Nitric oxide blocks the development of the human parasite *Schistosoma japonicum*. P Natl Acad Sci USA. 2017;114(38):10214-19.

MINOR CRITIQUE

For Fig 6, panels G and H, the authors should consider whether a Spearman rank correlation would be more appropriate for analysis, rather than the Pearson correlation. It is unclear that TGF-beta 3 would be expected to have a parametric (linear) correlation with either fibrosis or alpha-SMA. If the relationship is non-linear, Spearman rank would be more appropriate and more sensitive to detect a correlation.

>> Thanks a lot for the comments. We have used the Spearman rank correlation to reanalyze the correlation between TGF-beta 3 and fibrosis (Revised Figure 6). We agree that the Spearman rank is more appropriate. And we have revised the relevant content in the manuscript (Line 384-387; 752-753).

The other experimental methods presented are appropriate and executed correctly. The sample sizes were appropriate. There are no ethical or regulatory concerns.
>>Thanks a lot for the positive comments.
--------------------

**Results**


-Does the analysis presented match the analysis plan?

-Are the results clearly and completely presented?

-Are the figures (Tables, Images) of sufficient quality for clarity?


Reviewer #1:

1.Authors wrote ‘When infected with a low dose of parasites, no death was found both in WT or KO rats...', what are the bases for choosing the low or high doses of parasites？

>> Many thanks for the comments. We are sorry that we didn't clearly describe the result in the previous manuscript. To study the immunity and susceptibility of iNOS-KO rats in this paper, we used the infection dose of 200 cercariae per rat (low dose), which was the commonly used dose of infection in rat studies (*Exp. Parasitol.,2012,131(2):180-9; PNAS, 2017,114(38); Exp. Parasitol.,1979,47(2):232-45.*), but no death was found both in WT or KO rats. Therefore, to observe the survival rate of WT and KO rats, we continued to use the dose of 1000 cercariae (high dose), which was commonly used as a high dose in rats and in the animals that are highly resistant to *Schistosoma* infection (*Exp. Parasitol.,2012,131(2):180-9; Exp. Parasitol.,1979,47(2):232-45.*). We have updated the description of this result in the manuscript (Lines 188-193).

2.Authors wrote "This suggested an attenuation of T cell function and increased B cell function...". Actually, the author only showed the frequency of T cells and B cells.

>> Thanks a lot for the comment. In this manuscript, we have detected the production of effector cytokines of distinct T subsets, including Th1-related effector cytokines IFN-γ, TNF-α, and IL-2, Th2-related effector cytokines IL-4, IL-5, and IL-13. However, as you kindly mention, we have not further studied the direct function of these effector cytokines and B cells in response to *S. japonicum* infection in rats, and we have deleted the inaccurate descriptions and revised the content (Lines 207-208).

3.Authors wrote "To evaluate the role of iNOS in regulating the host’s T cell immune function...". In fact, besides αβ T lymphocytes（CD4 and CD8 T cells), γδ T lymphocytes are unconventional immune cells, which have both innate- and adaptive-like features allowing them to respond to a wide spectrum of pathogens. I think the results of the frequency and function of γδ T lymphocytes in rats during Schistosoma japonicum infection should be provided.

>> Thanks for the comment and the suggestion. γδ T lymphocytes have been reported in humans and animals, which play critical bridge-features both in innate and adaptive responses. However, γδ T cells represent an unconventional subset of T lymphocytes, no more than 1% in blood, spleen and LNs in rat (Ref: Res. Vet. Sci., 2012,93(1):168-71.). Rats have been reported to resistance to *Schistiosoma* infection mainly due to T cells and adaptive immunity (Ref*: 1. PLoS Negl Trop Dis, 2020, 14(12):e0008909; 2.* *J Immunol, 1991;147(11):3967-72*.). Furthermore, some published studies found that iNOS deficiency have influenced the function of CD4^+^ and CD8^+^ T cells in the mouse infection model including schistiosomiasis (Refs*: 1. PLoS Pathog, 2016,12(2):e1005442; 2. Infect Immun,1998, 66(8):3510-8; etc.*), and some have discussed the effect of exogenous or endogenous NOS2-derived NO on mouse and human T cells and B cells but only a few studies have focused on γδ T cells (Ref*:* *Trends Immunol, 2015;36(3):161-78.*). Taken together, we focused on the αβ T cells and B cells mediating the adaptive immune response to *Schistosoma* infection in the current study. However, it's very important to understand the immunological characteristics and functions of γδ T cells in *Schistosoma* infection, especially in the rat, and of course more studies on this field should be carried out in the future.

4.Please provide the statistics results of immunohistochemistry.
>> Thank you for reminding us. We have added the statistical results of immunohistochemistry in the revised manuscript (Revised Figure 5C).

Reviewer #2: MINOR CRITIQUES

The scanning EM results in Fig S2 are only presented in Pg 15 of the Discussion section, however the findings from these studies should be presented in the Results section, probably in the section of text discussing Fig 1 (pg 7).
>> Thanks for the critical suggestions. We have added the scanning EM results in the Results section as follows (Lines 174-178): “when we compared the development of *S. japonicum* in WT and iNOS-KO rat, the results of SEM showed that most of the spines in the middle part of the body of the schistosomula from iNOS-KO rats had disappeared. This is similar to what is observed in infected mice, while large numbers of spines on the tegument were still present in the middle part of the body of the schistosomula from WT rats (Fig. S1).”

Fig 5A: The bracket annotations showing significant p-values comparing different bars on the bar graph are missing for Eotaxin-2 and CXCL1.

>> Thanks a lot for the comment and the kind reminder. We have added the annotations of the statistical analysis results of Eotaxin-2 and CXCL1 in the Revised Figure 5.

--------------------

**Conclusions**


-Are the conclusions supported by the data presented?

-Are the limitations of analysis clearly described?

-Do the authors discuss how these data can be helpful to advance our understanding of the topic under study?

-Is public health relevance addressed?


Reviewer #1: I have no comments.

Reviewer #2: A major weakness throughout this paper is that, at several points, the authors report causal links between iNOS, immune cell subsets, and protection against parasite infection, however these conclusions are not supported by the data presented and the limitations of their analyses are not adequately described. There are multiple examples where the conclusions overstep the data and/or the effect of the iNOS knockout is described too vaguely:

Pg 9: “Thus, the results reveal that NO is necessary for maintaining the host’s cellular immune defense against infection in wild-type rats.”
Pg 17: “NO is necessary for maintaining the host’s cellular immune defense against S. japonicum in wild-type rats.”
Fig 2 only shows that iNOS-KO rats have different frequencies of B cells, T cells, and T cell subsets during infection, but this does not equate to NO being necessary for “cellular immune defense against infection.” Furthermore, in Panel E, there are no significant differences between infected WT and infected KO rats in CD4/CD8 ratio, so there is no evidence for a relationship between CD4/CD8 ratio and protection against schistosome infection. To demonstrate that iNOS is necessary for cellular immunity, as claimed, the knockout phenotype needs to be rescued experimentally by adoptive transfer, as outlined in my Methods critique above.
>>Thanks for the critical comments. We have deleted these inaccurate descriptions and revised the content (Lines 207-212). We further reviewed the related literature and found that in the schistosomiasis infection model, the CD4/CD8 ratio seems to be not a very useful indicator for evaluating cellular immune function. For the fluency of the full text, we have deleted this content.

To demonstrate iNOS which is necessary for maintaining the host’s immune activity, we have added the rescued experiment by adoptive transfer as suggested (Lines 295-302; Lines 328-331; Figure 6). We hope that the reviewer can feel comfortable with our response to these critical comments.

Pg 11: “The results suggest that high expression levels of iNOS are required to sustain CD4+ T cell responses in rats with schistosomiasis.” This claim is not supported by the preceding data in Fig 4. In panels B and D, in infected rats there are no significant differences between WT and KO in % IL-4 positive or % IFN-g positive CD4 T cells. Thus, the absence of iNOS had no effect on the frequency of IL-4 or IFN-g-producing CD4 T cells during schistosome infection.
>>Thanks for the critical comment. We have revised the content (Lines 288-304). In Figure 4A-4D, the leukocytes from WT and KO rats were stimulated with polyclonal PMA plus ionomycin PMA, and the IFN-γ or IL-4 positive CD4^+^ T cells were not all schistosome antigen-specific, but it also represented an effector ability of T cells. To confirm this conclusion, the production of IFN-γ and IL-4 in splenocytes culture supernatants stimulated for 48 hours with schistosome worm antigens (SWA) was also detected. The result showed that the production of IFN-γ and IL-4 in splenocytes isolated from infected KO rats was significantly lower than that isolated from infected WT rats (Revised Fig. 4E and 4F), suggesting that Th1 and Th2 response were decreased in the absence of iNOS. The results are as below:

Furthermore, when adoptive transfer of wild-type macrophages was performed in KO rats, the production of IFN-γ and IL-4 in splenocytes stimulated with SWA was significantly increased *in vitro* compared with non-transferred controls (Revised Fig. 6). The results are as below:

Therefore, these results suggested that high expression levels of iNOS ensured maximal development of Th2 and Th1 responses in rats with schistosomiasis.

Pgs 11-12: The authors report the strikingly discordant results in IL-6 expression between the serum and the liver, however they do not comment on why this discordance exists and its potential implications of immunity to S. japonicum.

>> Thanks for the comment and kind reminder. We have added the comments of this result in the Discussion section of this manuscript (Lines 541-547) as follows:

“It is worth noting that the expression of IL-6 in serum is inconsistent with that in liver. IL-6 is also an inflammatory cytokine and it was elevated in the serum of iNOS-KO rats. It is considered that this might be derived from several different damaged organs besides the liver reflecting the overall level of inflammation in the host, and this might be attributed to the significantly increased parasite burden and development-mediated inflammation.”

Pg 13: The authors use the results from the schistosome egg-induced pulmonary granuloma model to claim that “the exacerbated granuloma-associated inflammation and fibrosis in the liver of iNOS-KO rats were attributed to the increased worm burden, egg load, and viability, but not the effects of iNOS on pathology.” This claim assumes that the mechanisms that mediate fibrosis in the lung and the liver are similar, however this is not the case. The mechanisms of fibrosis are quite tissue-specific, and cannot be analogized between different tissues. For instance, cirrhosis is a fibrotic mechanism that is unique to the liver, and does not occur in any other tissue. Thus, the effects of iNOS on worm burden, egg load, and viability may be due to liver-specific effects that cannot be recapitulated in a pulmonary granuloma model, so lung fibrosis data cannot be extrapolated to the liver context.
>>Thanks for the critical comment. We agree that the mechanisms of fibrosis are quite tissue-specific. We are sorry that we haven’t been precise in describing the conclusion. Therefore, we have deleted the inaccurate description (Line 364-367).

Pg 15: “S. japonicum-elicited a Th2 response that was significantly impaired in iNOS-KO rats. Therefore, this study demonstrates that the difference in iNOS levels between mice and rats is responsible for the different immune responses and outcomes induced by schistosome infection in both hosts.” This is an incomplete interpretation of the data, as Th1 responses were also significantly impaired—specifically IFN-g—both systemically (Fig 3B) and in the liver (Fig 5A). Indeed, the authors need to expand on their interpretation of why iNOS-KO leads to suppression of both Th1 and Th2 cytokines, and to be more precise in how this affects existing models of the Th1/Th2 balance in anti-schistosomal immunity.

>>Thanks for the constructive comments and suggestions. We have modified the sentence (Lines 402-405) and added the interpretation of this result in the Discussion section (Lines 469-496) as follows:

CD4^+^ T cell-mediated Th1 and Th2 responses play an important role in against infection and immunopathogenesis of schistosomiasis. A strong Th1 response is highly desirable for an effective host defense in the early stage of infection and involved in inflammation, whereas Th2 response exerts anti-inflammatory effects and regulates Th1-mediated immunopathology [30, 31, 34, 40]. Our results showed that compared with the infected WT rats, the frequency of IL-4-producing CD4^+^T cells was impaired in the spleen and the levels of Th2-associated cytokines (IL-4 and IL-5) in the serum and liver were remarkably reduced in the infected iNOS-KO rats. Surprisingly, the reduction in Th2 response did not result from an increased Th1 response, because the production of Th1-related cytokines in the serum, spleens, and liver of infected iNOS-KO rats were also obviously decreased, in comparison to the infected WT rats. Previous studies reported that NO preferentially induced Th1 cell differentiation and Th1-related cytokines production by selectively up-regulating IL-12 receptor expression via cGMP [41]. This suggests that when NO is present in WT rats, NO may preferentially induce a Th1 response stimulated by schistosome antigens in the early stage of infection and participates in the anti-schistosome response. The continuously elevated Th1 response driven by NO, on the one hand, inhibits the survival of *Schistosoma*, and on the other hand, will initiate the host protective mechanism, that is, induce the increase of Th2 response to inhibit the excessive Th1 pro-inflammatory response. However, in the absence of inducible NO in iNOS-KO rats, the Th1 response may not be effectively induced, and the host may not be able to start the Th2 response without an excessive Th1 response, which leads to the inability of *Schistosoma* to be killed. Therefore, these results indicate that iNOS expression ensures maximal development of Th2 and Th1 responses in the rat during *Schistosoma* infection.

Furthermore, there is no data in this paper comparing immunity and infection outcomes between mice and rats, so the second claim is unsupported.

>> Thanks for the comment. Although there is no data in this paper comparing immunity and infection outcomes between mice and rats, we have summarized the differences of immunity and infection results between mice and rats infected with *Schistosoma* reported by others in the Introduction (Lines 84-105) and Discussion (Lines 391-396) section of the article. In addition, in our earlier published articles (*PNAS, 2017, 114(38):10214-9; PLoS One. 2012;7(4):e35834; PLoS One. 2013;8(5):e63650*), we have compared the infection results and NO concentration as well as the iNOS expression between mice and rats. All the results showed that the expression of iNOS and the levels of NO in rats was significantly higher than that in mice, and the worm load and development in mice were significantly higher than that in rats. Furthermore, previous studies showed that infected iNOS-KO mice developed increased Th1-associated cytokine expression and lower expression of Th2-associated cytokine levels, and displayed partially reduced resistance (*Infect Immun. 1998;66(8):3510-8.*), which differed from the observations obtained from infected iNOS-KO rats. Therefore, we feel justified in speculating that “the difference in iNOS levels between mice and rats may be responsible for the different immune responses and outcomes induced by schistosome infection in both hosts”. We hope that the reviewer now agrees.

Pg 15: The authors interpret the data from Fig 1A as implying “that it is in the liver rather than in the lungs that NO is effective in eliminating the parasite.” However, the authors do not suggest any potential mechanism to explain the early parasite clearance seen at the 1 week time point (albeit of a smaller magnitude than the clearance seen at 7 weeks).
>>Thanks again for the comments. As a cytotoxic agent, although NO is more effective in eliminating the parasites in the liver, it also has partial killing effect in the lung. Thus, the mechanism of the early parasite clearance at the 1 week time point was also attributed to NO. We have modified the sentence as follows (Lines 421-426):

The result implies that NO, as a cytotoxic agent, is more effective in eliminating the parasites in the livers than in the lungs, which may be due to the fact that NO can also function as a vascular relaxing factor to influence the ability of schistosomes to negotiate the vascular beds of the lungs so as to the migrating schistosomula can partially protect against the cytotoxic actions of NO.

Pg 19: “...the expression of iNOS accelerates the pulmonary granuloma-associated fibrotic process in rats.” This interpretation contradicts the data in Fig 6B, which suggests that iNOS actually impairs early fibrosis (7 days) but shows no significant difference between WT and KO by 14 days.
>>Thanks for the comments. The pulmonary granuloma-associated fibrotic process mentioned here refers to the development of fibrosis in the period from 7 days to 14 days, which displayed a slight increase in collagen deposition in WT rats and significant decreased in KO animals at day 14 compared with day 7 post-challenge. We believe we have adequately explained it in the manuscript as follows (Lines 352-355): Furthermore, in comparing the fibrotic process in KO and WT rats, WT rats displayed a slight increase in collagen deposition at day 14 compared with day 7 post-challenge. In contrast, collagen deposition was markedly decreased in KO animals at day 14, with nearly 56% less than that found at day 7 post-challenge (*P*＜0.01) (Fig. 7B). We hope the reviewer agrees.

The authors do not discuss a major limitation of the the iNOS-KO model—it is unclear which iNOS-expressing tissues or cell subsets are the primary drivers of the S. japonicum susceptibility phenotype. This could be addressed using tissue-specific and/or inducible knockout systems, but at a minimum, this limitation should be stated in the discussion.
>> Thanks for the comment and suggestions. We have added the limitations of the iNOS-KO model in the section of Discussion in the revised manuscript as follows (Lines 598-601):

However, using the systemic knockout iNOS gene rat model, rather than a tissue-specific or cell-specific conditional knockout model, we cannot determine which iNOS-expressing tissues or cell subsets are the primary drivers of the *S. japonicum* susceptibility phenotype.

Finally, the Discussion needs to focus more on potential mechanisms by which iNOS exerts its effects. Upon what molecules is NO acting in this context? What are the signaling pathways activated by NO that may lead to the observed immune phenotypes?
>> Thanks for the critical comment and suggestions. Regarding the potential mechanisms and signaling pathways of NO actions as the reviewer asked, we believe that they are very complex processes. As an active small molecule, NO has many targets. Most of the current research on NO are based on mouse models. However, a large amount of evidence indicates that the effects of NO in the immune system vary with host species and concentration. Therefore, the relevant mechanisms and signaling pathways seem to be difficult to apply to rats. In fact, we are currently trying to study the molecular mechanisms and signal pathways of NO in rats particularly when they are infected with parasites form protozoan to helminths. However, the research conditions when using rats are not as easy to use as those of mice, with, for example, the lack of many antibodies or transgenic animal models. These studies have turned out to be much more difficult than we thought. At present, therefore, we have obtained few further positive results for *S. japonicum* infection. Nevertheless, we believe that the *iNOS^-/-^* rat model can provide many novel results useful for our academic communities. For instance, Wang ZJ and his colleagues published their results with the iNOS KO rat infected with *Toxoplasma gondii* in iScience (2021) in which they found that the *iNOS^-/-^*-SD rat were completely resistant to *T. gondii* infection. Once the *iNOS^-/-^* rat peritoneal macrophages (PM) were infected with *T. gondii*, they produced high levels of reactive oxygen species (ROS) triggered by GRA43 secreted by *T. gondii*, which damaged the parasitophorous vacuole membrane and PM mitochondrial membranes within a few hours post infection. Further evidence indicated that the high levels of ROS caused mitochondrial superoxide dismutase 2 depletion and induced PM pyroptosis and cell death. This discovery of complete resistance to *T. gondii* infection, in the *iNOS^-/-^*-SD rat, demonstrates a strong link between NO and ROS in immunity to *T. gondii* infection and showcases a novel and effective backup innate immunity system. (Wang ZJ et al., 2021, iScience, online)
--------------------

**Editorial and Data Presentation Modifications?**


Use this section for editorial suggestions as well as relatively minor modifications of existing data that would enhance clarity. If the only modifications needed are minor and/or editorial, you may wish to recommend “Minor Revision” or “Accept”.

Reviewer #1: The authors report the immunomodulating activities of iNOS in rats, during S. japonicum infection, which leads to a rapid rejection of schistosome survival and no granuloma formation. Some interesting data were obtained in tests of the immuno assays and anti-granuloma formation activity.
However, upon initial review, several aspects are lacking and require revision before it can be considered further.

>>We are greatly appreciated the positive comments and kindly suggestions. We have tried our best to revise the manuscript.

Reviewer #2: There are multiple grammatical and typographical errors that need to be reviewed and corrected.

>>Many thanks for the comments and kind advice. They are very helpful for us to improve our paper. We have tried our best to revise the manuscript. We appreciate the efforts and hope that our revisions meet with your approval.

--------------------

**Summary and General Comments**


Use this section to provide overall comments, discuss strengths/weaknesses of the study, novelty, significance, general execution and scholarship. You may also include additional comments for the author, including concerns about dual publication, research ethics, or publication ethics. If requesting major revision, please articulate the new experiments that are needed.

Reviewer #1: (No Response)

Reviewer #2: This manuscript by Shen et al. describes an iNOS knockout rat model of Schistosoma japonicum infection. The authors demonstrate that iNOS deficiency enhances S. japonicum parasite burden and disease pathology and describe differences in lymphocyte subsets as well as cytokine and chemokine expression associated with iNOS knockout. Strengths of the study include the detailed characterization of the immune and tissue phenotypes of the knockout rat, particularly when the rats are infected. Novel findings include the co-regulation of both Th1 and Th2 responses by iNOS during S. japonicum infection as well as varied roles for iNOS in first suppressing and then enhancing fibrosis in a pulmonary granuloma model. However, the paper fails to present experiments that establish direct causal links between iNOS and its necessity for cellular immunity that mediates protection against S. japonicum. I am further concerned that throughout the manuscript, the authors draw several key conclusions that are either vaguely worded or unsupported by their data. While I describe major additional experiments that could be performed to establish the causal links that the authors claim, I think that for the authors’ interpretations to align more precisely with the data they present will require a major reorganization and rewrite of the manuscript. For those reasons, I recommend to reject the manuscript at this time.

>>Thank you for the profound comments. We acknowledge that there are shortcomings in the current format of our manuscript, and we have tried our best to improve it. Thus far, little is known in rats about the regulatory role of NO in the immune system and the outcomes of iNOS gene deficiency on the evolving immune response to *S. japonicum* infection but interesting results were published with *iNOS^-/-^* rats infected with *T. gondii* (Wang ZJ et al., iScience, 2021). We believe that although there are many more difficulties associated with the rat model system than those with mice when studying the immune response to parasite infection, the more work that can be done with the *iNOS^-/-^* rat model, the more novel results there will be obtained to explain the mechanisms for the immune responses when they are infected with *S. japonicum*. Nevertheless, we believe the results obtained in this study are of great reference value in the study of rat immunity and NO when infected with *S. japonicum*.
--------------------

PLOS authors have the option to publish the peer review history of their article ([what does this mean?](https://journals.plos.org/plosntds/s/editorial-and-peer-review-process#loc-peer-review-history)). If published, this will include your full peer review and any attached files.


If you choose “no”, your identity will remain anonymous but your review may still be made public.


**Do you want your identity to be public for this peer review?** For information about this choice, including consent withdrawal, please see our [Privacy Policy](https://www.plos.org/privacy-policy).

Reviewer #1: No

Reviewer #2: No
