# Supplementary material for: iNOS is essential to maintain a protective Th1/Th2 response and the production of cytokines/chemokines against Schistosoma japonicum infection in rats
Source: PLoS Negl Trop Dis. 2022 May 18;16(5):e0010403. doi: 10.1371/journal.pntd.0010403 (PMC9116669; doi:10.1371/journal.pntd.0010403)
Supplement: Attachment — Submitted filename: The letter Response to Review comments.docx [file pntd.0010403.s006.docx]

Dear Reviewers,

Thank you for the review of our manuscript “iNOS is essential to maintain host immune activity against Schistosoma japonicum infection in rats”. We appreciate the constructive comments from you, which helped us to increase the clarity of the manuscript and interpret our data more accurately. We have tried our best to revise the manuscript. Please, see attached the point-by-point replies to the comments and the edited version of the manuscript with tracked changes.

We appreciate the efforts of you and hope that our re-submission will meet your expectations to be accepted for publication.

With the best wishes.

Yours sincerely,

Zhao-Rong Lun, PhD

On behalf of all co-authors

Professor in Zoology/Parasitology

State Key Laboratory of Biocontrol

Sun Yat-Sen University

Guangzhou 510275, P.R. China

Email: lsslzr@mail.sysu.edu.cn

**Methods**


-Are the objectives of the study clearly articulated with a clear testable hypothesis stated?

-Is the study design appropriate to address the stated objectives?

-Is the population clearly described and appropriate for the hypothesis being tested?

-Is the sample size sufficient to ensure adequate power to address the hypothesis being tested?

-Were correct statistical analysis used to support conclusions?

-Are there concerns about ethical or regulatory requirements being met?


Reviewer #1: No comment.

Reviewer #2: All my methodological critiques have been addressed, particularly with the experiments employing adoptive transfer of WT macrophages.
>>We are greatly appreciated the positive comments and kind suggestions.

--------------------

**Results**


-Does the analysis presented match the analysis plan?

-Are the results clearly and completely presented?

-Are the figures (Tables, Images) of sufficient quality for clarity?


Reviewer #1: No comment.

Reviewer #2: See comments under "Conclusions".

--------------------

**Conclusions**


-Are the conclusions supported by the data presented?

-Are the limitations of analysis clearly described?

-Do the authors discuss how these data can be helpful to advance our understanding of the topic under study?

-Is public health relevance addressed?


Reviewer #1: No comment.

Reviewer #2: Several related concerns regarding reporting of results and interpretation of Figure 4:

Lines 262-264: “As shown in Fig.4A and 4B, the frequency of CD4+ T cells producing IL-4 was slightly increased in the blood (P<0.05) of naïve KO rats, compared with naïve WT rats.” Fig 4B shows no significant difference in %IL-4+ CD4 T cells between WT naïve and KO naïve. Please correct this text.

>> Thanks for your kind reminder. We are sorry for the carelessness and we have corrected in the manuscript (Lines 262-264).

Lines 266-270: “However, although the frequency of IL-4-producing CD4+ T cells slightly increased in infected KO rats, when compared to naïve KO rats, no significant differences were found in LN and spleens (P>0.05) although these were lower than those found in the infected WT rats.” The phrasing here is unclear – Fig 4B shows that in KO animals, only blood showed a significant increase between naïve and infected, however this is not specified in the text. Also when the authors state “these were lower than those found in the infected WT rats” it seems they are saying that the *magnitude* of increase between naïve and infected is higher in the WT than in the KO animals. Please clarify the language reporting these results.

>>Thanks for the comments and suggestions. We have clarified the language reporting these results in the revised manuscript (Lines 267-270).

Line 270: “The results suggest an impaired Th2 response in infected iNOS-KO rats.” I do not think the results in Fig 4 support this interpretation; although the magnitude of the increase in %IL-4+ or %IFN-gamma between naïve and infected is higher in WT than in KO rats, the actual percentage of IL-4+ or IFN-gamma CD4 T cells is not significantly different between infected WT and infected KO rats (except for IL-4+ in the blood, which decreases, as shown in Fig 4B). So because the % of cytokine positive CD4 T cells is not different between infected WT and infected KO, I don’t think you can argue that these results alone indicated an impaired Th2 (or Th1) response. In contrast, Fig 4E and 4F provide much stronger evidence showing that both Th1 and Th2 responses are impaired in KO animals.

>> Thanks a lot for the comments. We have revised the descriptions in the revised manuscript (Lines 270-285).

Lines 270-274: “The reduction in the Th2 response did not result from an increased Th1 response because the frequency of CD4+ T cells producing IFN-γ was also decreased in the blood, LN, and spleens of infected KO rats, compared with infected WT rats (P<0.05) (Fig.4C and 4D).” Similar problem as with Lines 266-274 – there are no significant differences between infected WT and infected KO rats in %IFN-g CD4 T cells in blood, LN, or spleen. Naïve animals show lower %IFN-g CD4 Ts in KO vs WT for LN and spleen, but not blood, and the magnitude of increase in %IFN-g CD4 Ts between naïve and infected is smaller for KO vs WT in blood, but is actually higher in KO vs WT in LN and spleen. Please be more precise in accurately reporting these data in the Results section.
>> Thanks a lot for the comments. We have modified the descriptions of these data in the revised manuscript (Lines 270-277).

Figure 8: I recommend revising the part of the schematic showing that CD3+ and CD4+ T cells decrease with infection when iNOS is present, but that these effects are reversed when iNOS is absent, as this is not supported by the data in Fig 2. In Fig 2B, infection of WT rats does show a non-significant trend towards decreasing T cell %, but this trend does not reverse when KO rats are infected, so I don’t think this warrants inclusion in the summary schematic. In contrast, in Fig 2D in the spleen, CD4 T cells go down when WT rats are infected, but increase when KO rats are infected, so this does fit the current version of the schematic, however this trend does not reach statistical significance for the LN and is not present in the liver, so the schematic should be revised to reflect this accurately.
>>Thanks for the comments and suggestions. We have revised the schematic in the revised Figure 8.

--------------------

**Editorial and Data Presentation Modifications?**


Use this section for editorial suggestions as well as relatively minor modifications of existing data that would enhance clarity. If the only modifications needed are minor and/or editorial, you may wish to recommend “Minor Revision” or “Accept”.

Reviewer #1: No comment.

Reviewer #2: In the Financial Disclosure, the authors need to submit a statement explicitly addressing whether sponsors or funders played “any role in the study design, data collection and analysis, decision to publish, or preparation of the manuscript.”
>> Thanks for the suggestions. We have submitted a statement explicitly addressing the role of each fund in preparing this article in the Financial Disclosure.

There remain multiple grammatical and typographical errors that will need to be corrected prior to publication.
>> Thanks for the comments. We have checked and corrected all grammatical and typographical errors.

--------------------

**Summary and General Comments**


Use this section to provide overall comments, discuss strengths/weaknesses of the study, novelty, significance, general execution and scholarship. You may also include additional comments for the author, including concerns about dual publication, research ethics, or publication ethics. If requesting major revision, please articulate the new experiments that are needed.

Reviewer #1: No comment.

Reviewer #2: This revised manuscript by Shen et al. employs an iNOS knockout rat model to illustrate the key roles of iNOS and NO signaling in immunopathology and protective immunity to Schistosoma japonicum. The authors’ revisions have substantially improved the quality of the manuscript, particularly by (1) demonstrating that adoptive transfer with WT macrophages partially rescues several of the immune phenotypes, (2) using more precise language in reporting their research findings, and (3) delineating more clearly in their Discussion between interpretations that are firmly supported by the data versus more speculative hypotheses that provide the foundation for future studies. My remaining critiques are comparatively minor and focus on more precise reporting and interpretation of the results in Figure 4, as well as revision of the summary schematic in Figure 8 to more accurately reflect the data on T cells and CD4 T cells reported in Figure 2. With these revisions, coupled with the thorough characterization of the iNOS-KO phenotype in Schistosoma japonicum infection, this has the potential to be an impactful manuscript for studies of mammalian immunity in schistosomiasis. I recommend Minor Revisions before the manuscript is accepted for publication.
>> Many thanks for the positive comments and kindly suggestions. We have tried our best to revise the manuscript. We appreciate the efforts and hope that our revisions meet with your approval.

--------------------

PLOS authors have the option to publish the peer review history of their article ([what does this mean?](https://journals.plos.org/plosntds/s/editorial-and-peer-review-process#loc-peer-review-history)). If published, this will include your full peer review and any attached files.


If you choose “no”, your identity will remain anonymous but your review may still be made public.


**Do you want your identity to be public for this peer review?** For information about this choice, including consent withdrawal, please see our [Privacy Policy](https://www.plos.org/privacy-policy).

Reviewer #1: No

Reviewer #2: No
