# Supplementary material for: iNOS is essential to maintain a protective Th1/Th2 response and the production of cytokines/chemokines against Schistosoma japonicum infection in rats
Source: PLoS Negl Trop Dis. 2022 May 18;16(5):e0010403. doi: 10.1371/journal.pntd.0010403 (PMC9116669; doi:10.1371/journal.pntd.0010403)
Supplement: Attachment — Submitted filename: Review Note for PNTD-D-21-00912_R2 Haroon Akbar.docx [file pntd.0010403.s007.docx]

**Review Note for PNTD-D-21-00912_R2**

The authors have presented massive amount of work and is highly appreciated.

Here are my observations:

- Suggestion for Title:

“**Knocking-out iNOS in rats disintegrates a balanced and protective Th1-Th2 immune response against *Schistosoma japonicum*, exacerbating acute pro-inflammatory response leading to host mortality**”

- Flow cytometric data displayed in Fig 2 B and 2 D histograms, is based on the percentage of different cell populations. I wish if the numbers of different cell populations can also be displayed in similar histograms to have an overview- how many times the immune cells have multiplied? Whether the numbers of these different cell populations, are also reflecting the same scenario as evident from their percentages? This could be really challenging but is not impossible. The starting volume of blood sample, the starting weight of liver, lymph nodes and spleen samples- can be greatly helpful in these calculations, to reach the overall cell numbers in the peripheral circulation as well as in different organs.
- Line 449-450: Please delete “in” [CD4+ T cell-mediated Th1 and Th2 responses play an important role ~~in~~ against infection and immunopathogenesis of schistosomiasis]
- Line 654: Please correct the sign of micron.

For Editor:

- No doubt, the knocking iNOS out in the rats, have led to exacerbation of the immune response with all the evidences supporting this hypothesis, the exact mechanism by which absence of iNOS has caused these effects, remains unexplored. This should be discussed in the discussion with a reflection in abstract and conclusion. The dramatic shift in the immune response from a complete protection to enhanced susceptibility certainly destabilizes multiple pathways. The authors have explored many cytokines in this regard. I wish if the authors would have also explored the kinetics of regulatory T cells (CD4+Foxp3+ with CD25+/- and CD8+Foxp3+ with CD25+/-) as these cells have been found to maintain an equilibrium while moderating the severe pro-inflammatory responses in the body, thus protecting host from the immunopathology. Same is known of C57-Black-6 mice that die of immunopathology during an infection with a protozoan parasite “*Toxoplasma gondii*”.

Performing a major supplementary experiment could be really embarrassing after second revision cycle, the authors may add this aspect in the discussion and reflect this aspect for their upcoming studies i.e. to sort out the effect of iNOS-deletion on regulatory T cells

- Total number of rats (both WT and KO) sacrificed for this study may please be mentioned and an estimation, in the ethics statement, may please be done to predict for future studies- to what extent this number may be reduced for such type of extensive studies.

Best wishes

haroon
