# Supplementary material for: iNOS is essential to maintain a protective Th1/Th2 response and the production of cytokines/chemokines against Schistosoma japonicum infection in rats
Source: PLoS Negl Trop Dis. 2022 May 18;16(5):e0010403. doi: 10.1371/journal.pntd.0010403 (PMC9116669; doi:10.1371/journal.pntd.0010403)
Supplement: Attachment — Submitted filename: The letter Response to Review comments.docx [file pntd.0010403.s008.docx]

Dear Reviewers,

Thank you for the review of our manuscript “iNOS is essential to maintain host immune activity against *Schistosoma japonicum* infection in rats”. We thank you for your comments and suggestions which significantly improved the manuscript. We are pleased to enclose the revised manuscript and point-by-point responses to the comments.

We appreciate your efforts and hope that our re-submission will meet your expectations to be accepted for publication.

With the best wishes.

Yours sincerely,

Zhao-Rong Lun, PhD

On behalf of all co-authors

Professor in Zoology/Parasitology

State Key Laboratory of Biocontrol

Sun Yat-Sen University

Guangzhou 510275, P.R. China

Email: lsslzr@mail.sysu.edu.cn

**Methods**


-Are the objectives of the study clearly articulated with a clear testable hypothesis stated?

-Is the study design appropriate to address the stated objectives?

-Is the population clearly described and appropriate for the hypothesis being tested?

-Is the sample size sufficient to ensure adequate power to address the hypothesis being tested?

-Were correct statistical analysis used to support conclusions?

-Are there concerns about ethical or regulatory requirements being met?


Reviewer #2: (No Response)

Reviewer #3: (No Response)

Reviewer #4: (No Response)

--------------------

**Results**


-Does the analysis presented match the analysis plan?

-Are the results clearly and completely presented?

-Are the figures (Tables, Images) of sufficient quality for clarity?


Reviewer #2: (No Response)

Reviewer #3: (No Response)

Reviewer #4: (No Response)

--------------------

**Conclusions**


-Are the conclusions supported by the data presented?

-Are the limitations of analysis clearly described?

-Do the authors discuss how these data can be helpful to advance our understanding of the topic under study?

-Is public health relevance addressed?


Reviewer #2: (No Response)

Reviewer #3: (No Response)

Reviewer #4: (No Response)

--------------------

**Editorial and Data Presentation Modifications?**


Use this section for editorial suggestions as well as relatively minor modifications of existing data that would enhance clarity. If the only modifications needed are minor and/or editorial, you may wish to recommend “Minor Revision” or “Accept”.

Reviewer #2: (No Response)

Reviewer #3: (No Response)

Reviewer #4: The authors have presented massive amount of work and is highly appreciated.
Here are my observations:
• Suggestion for Title:
“Knocking-out iNOS in rats disintegrates a balanced and protective Th1-Th2 immune response against Schistosoma japonicum, exacerbating acute pro-inflammatory response leading to host mortality”
>>Thanks for the kind suggestion. We think the title may be more appropriate: iNOS is essential to maintain a protective Th1/Th2 response and the production of cytokines/chemokines against *Schistosoma japonicum* infection in rats. We hope the reviewer agrees.

• Flow cytometric data displayed in Fig 2 B and 2 D histograms, is based on the percentage of different cell populations. I wish if the numbers of different cell populations can also be displayed in similar histograms to have an overview- how many times the immune cells have multiplied? Whether the numbers of these different cell populations, are also reflecting the same scenario as evident from their percentages? This could be really challenging but is not impossible. The starting volume of blood sample, the starting weight of liver, lymph nodes and spleen samples- can be greatly helpful in these calculations, to reach the overall cell numbers in the peripheral circulation as well as in different organs.
>> Thanks for the suggestions. We have carefully checked our manuscript and we don't think that these actions are very helpful to improve the quality of the manuscript, and these contents are not the focus of this paper. Therefore, we sincerely hope that the reviewer can understand our suggestions.

• Line 449-450: Please delete “in” [CD4+ T cell-mediated Th1 and Th2 responses play an important role in against infection and immunopathogenesis of schistosomiasis]

>> Thanks for the suggestions. we have done it.

• Line 654: Please correct the sign of micron.

>> Thanks again for your kind reminder. We have checked and corrected it.

--------------------

**Summary and General Comments**


Use this section to provide overall comments, discuss strengths/weaknesses of the study, novelty, significance, general execution and scholarship. You may also include additional comments for the author, including concerns about dual publication, research ethics, or publication ethics. If requesting major revision, please articulate the new experiments that are needed.

Reviewer #2: (No Response)

Reviewer #3: This manuscript may be suitable for acceptance as the comments of the previous reviewers have been well addressed. My only suggestion is that the resolution of the figures, especially the histo-immunostaining pictures, needs to be improved to show the area indicated by the arrow more clearly.

>>Thanks for the comment and suggestion. We have uploaded high-resolution figures. The lack of clarity in the current version may be due to the status under review, we believe that the figures will be clear enough in the future published version.

Reviewer #4: (No Response)

--------------------

PLOS authors have the option to publish the peer review history of their article ([what does this mean?](https://journals.plos.org/plosntds/s/editorial-and-peer-review-process#loc-peer-review-history)). If published, this will include your full peer review and any attached files.


If you choose “no”, your identity will remain anonymous but your review may still be made public.


**Do you want your identity to be public for this peer review?** For information about this choice, including consent withdrawal, please see our [Privacy Policy](https://www.plos.org/privacy-policy).

Reviewer #2: No

Reviewer #3: No

Reviewer #4: Yes: Dr Haroon AKBAR, Faculty of Veterinary Science, University of Veterinary and Animal Sciences, Lahore, Pakistan
